# TLK1‐mediated MK5‐S354 phosphorylation drives prostate cancer cell motility and may signify distinct pathologies

**DOI:** 10.1002/1878-0261.13183

**Published:** 2022-02-03

**Authors:** Md Imtiaz Khalil, Vibha Singh, Judy King, Arrigo De Benedetti

**Affiliations:** ^1^ Department of Biochemistry and Molecular Biology LSU Health Sciences Center Shreveport LA USA; ^2^ Department of Pathology and Translational Pathobiology LSU Health Sciences Center Shreveport LA USA

**Keywords:** cell motility, MK5/MAPKAPK5/PRAK, PCa TMA, prostate cancer metastasis, TLK1/1B

## Abstract

Metastases account for the majority of prostate cancer (PCa) deaths, and targeting them is a major goal of systemic therapy. We identified a novel interaction between two kinases: tousled‐like kinase 1 (TLK1) and MAP kinase‐activated protein kinase 5 (MK5) that promotes PCa spread. In PCa progression, TLK1–MK5 signalling appears to increase following antiandrogen treatment and in metastatic castration‐resistant prostate cancer (mCRPC) patients. Determinations of motility rates (2D and 3D) of different TLK1‐ and MK5‐perturbed cells, including knockout (KO) and knockdown (KD), as well as the use of specific inhibitors, showed the importance of these two proteins for *in vitro* dissemination. We established that TLK1 phosphorylates MK5 on three residues (S160, S354 and S386), resulting in MK5 activation, and additionally, mobility shifts of MK5 also supported its phosphorylation by TLK1 in transfected HEK 293 cells. Expression of MK5‐S354A or kinase‐dead MK5 in MK5‐depleted mouse embryonic fibroblast (MEF) cells failed to restore their motility compared with that of wild‐type (WT) MK5‐rescued MK5^−/−^ MEF cells. A pMK5‐S354 antiserum was used to establish this site as an authentic TLK1 target in androgen‐sensitive human prostate adenocarcinoma (LNCaP) cells, and was used in immunohistochemistry (IHC) studies of age‐related PCa sections from TRAMP (transgenic adenocarcinoma of the mouse prostate) mice and to probe a human tissue microarray (TMA), which revealed pMK5‐S354 level is correlated with disease progression (Gleason score and nodal metastases). In addition, The Cancer Genome Atlas (TCGA) analyses of PCa expression and genome‐wide association study (GWAS) relations identify TLK1 and MK5 as potential drivers of advanced PCa and as markers of mCRPC. Our work suggests that TLK1–MK5 signalling is functionally involved in driving PCa cell motility and clinical features of aggressiveness; hence, disruption of this axis may inhibit the metastatic spread of PCa.

## Graphical abstract

Targeting metastasis in prostate cancer (PCa) remains a major challenge during systemic therapy. Here, we describe a novel interaction between the TLK1 and MK5 kinases, which resulted in activation and cytoplasmic relocalization of MK5 via MK5‐S354 phosphorylation. Knockout and pharmacological inhibition of MK5 impaired motility and invasion of PCa and MEF cells. Additionally, MK5‐S354 phosphorylation correlated with PCa progression in a TRAMP mouse model and correlated to disease progression in patients with PCa. Thus, disruption of the TLK1‐MK5 axis may inhibit the metastatic spread of PCa.

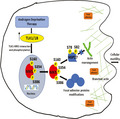

**Abbreviations**
ADT: androgen deprivation therapy
Co‐IP: co‐immunoprecipitation
CRPC: castration‐resistant prostate cancer
ECM: extracellular matrix
GS: gleason score
IHC: immunohistochemistry
IVK: *in vitro* kinase
LPP: lambda protein phosphatase
LSUHSC: Louisiana State University Health Sciences Center
mCRPC: metastatic castration‐resistant prostate cancer
MK5: MAPK‐activated protein kinase 5
MR: mean rate
MS: mass spectrometry
NEK1: NIMA‐related kinase 1
NLS: nuclear localization signal
PCa: prostate cancer
PIN: prostatic intraepithelial neoplasia
PRAK: p38‐regulated/activated kinase
SEM: standard error of the mean
THD: thioridazine
TLK1: tousled‐like kinase 1
TMA: tissue microarray
TSL: tousled kinase
uORF: upstream open reading frame
VC: vehicle control

## 1 Introduction

Prostate cancer (PCa) is the 2nd leading death causing malignancy in men in the United States [1]. Like other malignancies, metastasis has been the primary cause of PCa‐related deaths. PCa patients with distant organ metastasis have a 5‐year survival rate of only around 30% [2], and currently, no curative therapy is available for the treatment of metastatic PCa [3]. Androgen deprivation therapy (ADT) has been the gold standard method to treat PCa. However, after 1–2 years of treatment, the tumour relapses with more aggressive phenotype, known as metastatic castration‐resistant PCa (mCRPC). Cellular motility and invasion are the prerequisites for the cancer cells to migrate and localize in the adjacent and distant organs. Cell migration and invasion are multifactor processes involving numerous signalling pathways. Thus, identification of these factors and underlying signalling cascades is required to devise a therapeutic intervention, which may reduce the spread of the cancer cells.

Tousled kinase (TSL) was first discovered in *Arabidopsis thaliana* and found crucial for leaf and flower development of the plant [4, 5]. Later, homologues of TSL were identified in most metazoans, which are named as tousled‐like kinase (TLK) [6]. TLKs are serine/threonine kinases, with maximal activity in the S‐phase of the cell cycle, play roles in replication, transcription, chromosomal segregation, DNA damage response and repair. In mammals, two genes are present in the tousled‐like kinase family—*Tlk1* and *Tlk2*, which share 89% homology in their entire amino acid sequence and 94% similarity in their C‐terminal kinase domain (rev. in [7]). Each gene encodes several isoforms of which TLK1B, a spliced variant with long untranslated region and two regulatory uORFs, is well characterized by our group [8]. Histone H3, histone chaperone ASF1, RAD9 and NEK1 are some of the well‐validated substrates of TLK1, which can be directly phosphorylated by TLK1/1B. The activating phosphorylation of NEK1 on T141 residue by TLK1 results in the initiation of DNA damage response signalling through TLK1 > NEK1 > ATR > CHK1 axis that contributes to castration‐resistant progression of PCa [9, 10]. In addition, recent findings suggest that TLK1‐mediated activation of NEK1 also leads to apoptotic prevention and YAP stabilization, which facilitates cancer cell survival and conversion to androgen independence [11, 12]. Recently, two research groups independently reported that TLK2 enhances the migration rate and invasiveness of breast cancer and glioblastoma cell lines and hence, can be considered a factor for aggressiveness [13, 14]. Another study revealed that the gene encoding tousled‐like kinase (*Tlk*) in *Drosophila* (*Drosophila* has only one *Tlk* gene) is required for the collective migration of border cells by activating JAK/STAT signalling pathway in the ovary, a phenomenon that is reminiscent of cancer migration/metastasis [15]. Since TLK1 and TLK2 have higher sequence similarity and function similarly, we hypothesized that TLK1 may also play role in the dissemination of the cancer cells.

The 2nd protein player in this study is MAPK‐activated protein kinase 5 (MAPKAPK5 or MK5), a serine/threonine kinase that has been studied in several neoplastic and nonmalignant cells. MK5 can be activated by both atypical and typical upstream MAP kinase, ERK3/4 and p38, respectively. Hence, it has acquired the acronym PRAK (p38‐regulated/activated kinase). MK5 is known to be a genuine promotility factor that drives cell motility by regulating actin cytoskeletal rearrangements and focal adhesion complex modification [16, 17, 18, 19, 20, 21]. However, a few other studies suggested the antimotility role of MK5 in HeLa and U2OS osteosarcoma cells where MK5 exerts its function through the impairment of FAK activation and filamentous actin formation, respectively [22, 23]. MK5’s role in motility is thus dependent on the cellular context, type and stage of the cancer. Other biological functions of MK5 includes cell growth, oncogene‐induced senescence, transcriptional regulation, neuronal spine formation, cellular survival and regulation of cardiac fibroblast function [19, 24, 25, 26, 27, 28, 29, 30, 31, 32, 33]. Out of 11 members in the MAPKAPK family, MK5 is most closely related to MK2 and MK3 and shares approximately 33% sequence similarity (rev. in [34, 35, 36, 37]). Although MK2 and MK5 have several common substrates, their functions are temporally and spatially separated [17]. MK5 can act as both tumour suppressor and promoter [16, 25, 36, 38]. For instance, MK5 is reported to negatively regulate Myc transcription and positively regulate YAP’s stability by inhibiting its degradation [24, 39]. MK5’s implication in PCa metastasis has not been characterized yet.

Here, we describe the potential implications of TLK1‐MK5 signalling in prostate cancer cell motility and whether disruption of this axis can inhibit PCa cell migration. Our data suggest that MK5 is a direct substrate of TLK1, which can be phosphorylated on several serine residues. We also establish MK5 as a genuine promotility factor in PCa cell lines and suggest that TLK1 phosphorylation of MK5 may enhance its promotility activity. Our bioinformatic study also suggested a correlation of TLK1 and MK5 expression with the aggressiveness of prostate tumours, which hints towards a clinical relevance of TLK1‐MK5 signalling in PCa metastasis. In addition, IHC analysis of a PCa TMA revealed that high pMK5‐S354 stain correlated with the presence of local nodal involvement in biopsies.

## 2 Materials and methods

### 2.1 Plasmids and antibodies

Wild‐type human full‐length TLK1 mammalian expression plasmid was purchased from Addgene (Watertown, MA, USA, cat# 98378). Wild‐type full‐length human TLK1B bacterial expression plasmid was obtained from S. Kirubakaran, Discipline of Biological Engineering, Indian Institute of Technology, Gandhinagar, India [40]. Dominant negative human TLK1 kinase‐dead mammalian expression vector was generated as previously described [41]. pEGFP‐MK5‐C1 mammalian expression vector was a kind gift from O. Morten Seternes (Department of Pharmacology,1 Institute of Medical Biology, University of Tromsø, Tromsø, Norway) [42]. Human full‐length MK5 bacterial expression plasmid was purchased from Vector Builder (Chicago, IL, USA). The following primary antibodies were used in this study: rabbit anti‐TLK1 (Thermo Fisher, Waltham, MA, USA, cat# 720397), rabbit anti‐TLK1B (laboratory‐generated), rabbit anti‐MK5 (Cell Signaling Technology, CST, Danvers, MA, USA, cat# 7419S), mouse anti‐PRAK (Santa Cruz Biotechnology, SCBT, Dallas, TX, USA, sc‐46667), rabbit anti‐phospho‐MK5 S354 (Thermo Fisher, cat# PA5‐105676), mouse anti‐GFP (Thermo Fisher, cat# MA5‐15256), rabbit anti‐GAPDH (CST, cat# 2118S) and rabbit anti‐actin (Abcam, Cambridge, MA, USA, cat# ab1801).

### 2.2 Cell culture

Human embryonic kidney 293 (HEK 293) and mouse embryonic fibroblast (MEF) cells were cultured in DMEM supplemented with 10% FBS and 1% penicillin/streptomycin. PCa cell lines—LNCaP, C4‐2B, PC3, DU145 and 22Rv1—were cultured in RPMI 1640 supplemented with 10% FBS and 1% penicillin/streptomycin. VCaP cells were cultured in 1% fibronectin‐coated flask in DMEM supplemented with 10% FBS and 1% penicillin/streptomycin. MK5^−/−^ MEF cells were a kind gift from M. Gaestel, University of Hannover, Germany [43]. All the other cell lines were purchased from American Type Culture Collection (ATCC, Manassas, VA, USA), and all were authenticated within the past three years. All the cells were maintained in humidified incubator at 37 °C with 5% CO_2_.

### 2.3 Cell treatment

LNCaP cells were treated with 5 µm or 10 µm of bicalutamide (Selleckchem, Houston, TX, USA, cat# S1190) or 10 µm of thioridazine (THD; Sigma‐Aldrich, St. Louis, MO, USA, cat# T9025) or J54 [44] for 48 h in a 6‐well plate when the confluency reached 70‐90%. DMSO‐treated LNCaP cells were considered as vehicle control (VC). After the treatment, cells were harvested for western blotting (WB) analysis. GFP‐MK5 and TLK1 co‐expressing cell lysates were treated with lambda protein phosphatase (LPP) (New England Biolabs, Inc., Lincoln, NE, USA, cat# P0753S) following the manufacturer’s protocol.

### 2.4 Cell transfection

Wild‐type MEF (MEF WT) and LNCaP cells were transfected with eGFP‐MK5 by Lipofectamine 3000 (Thermo Scientific, Waltham, MA, USA, cat# L3000‐015) following the manufacturer’s protocol, and GFP‐positive cells were sorted by flow cytometry. MK5^−/−^ MEF cells were transfected with eGFP‐MK5 or TLK1 mammalian expression plasmid by Lipofectamine 3000 and sort‐gated by flow cytometry or selected by 1‐2 µg·mL^−1^ puromycin treatment for seven days. LNCaP cells were also transfected with kinase‐dead TLK1 plasmid and selected by 400–500 µg·mL^−1^ G418 for seven days. HEK293 cells were first transfected with eGFP‐MK5 and selected by flow cytometry. MK5‐positive cells were further transfected with either TLK1 or kinase‐dead TLK1 plasmid and selected by antibiotic treatment. To knock down TLK1, MEF WT cells were plated as 40 000 cells·well^−1^ in a 6‐well plate and grown until 60–70% confluency. Cells were transfected twice with 100 nm of mouse‐specific TLK1 siRNA (Thermo Scientific, Waltham, MA, USA, cat# AM16708) by Lipofectamine 3000. 72 h after the 2nd dose of siRNA treatment, cells were split and harvested for scratch wound repair assay and WB analysis.

### 2.5 Scratch wound repair assay

Different MEF derivatives and PCa (C4‐2B, PC3, DU145 and 22Rv1) cells were seeded as 40 000 cells·well^−1^ in a 96‐well ImageLock plate (Essen Biosciences, Inc., Ann Arbor, MI, USA, cat# 4379) in 10% FBS‐containing media. LNCaP cells were seeded as 80 000–90 000 cells·well^−1^ in a 96‐well ImageLock plate. After 48 h when 90–100% confluency was achieved, cells were scratched by a wound maker (Essen Biosciences, Inc., cat# 4493), washed twice with serum free media and supplemented with low serum (0.5‐1% FBS) media with or without treatment. Media were supplemented with either DMSO as vehicle control (VC) or 10 µm J54 [44] or 5‐20 µm of GLPG0259 (Medkoo Biosciences, Inc., Morrisville, NC, USA, cat# 561481). The plates were then transferred to a IncuCyte Zoom incubator with 5% CO_2_ at 37 °C, and the migration of the cells was monitored by IncuCyte Zoom live cell imaging system (Essen Biosciences, Inc., Ann Arbor, MI, USA). Machine‐controlled images were taken every 4 h to determine the relative wound density of each well. Mean rate (MR) was determined by calculating the slopes of the curve.

### 2.6 3D chemotactic trans‐well migration assay

Both sides of the porous membrane of the top insert of IncuCyte ClearView 96‐well chemotaxis plate (Essen Biosciences, Inc., cat# 4582) were coated with either fibronectin (1 mg·mL^−1^), or Matrigel (50 µg·mL^−1^), or collagen I (50 µg·mL^−1^) 3 h before seeding the cells. Cells were seeded in the top chamber as 1000 cells·well^−1^ in low serum (0.5% FBS) media. Chemotactic gradient was created by putting 10% FBS‐containing media in the bottom reservoir. Cells were transferred to a IncuCyte Zoom incubator with 5% CO_2_ at 37 °C, and the migration of the cells towards chemotactic gradient was monitored by IncuCyte Zoom live cell analysis system. Images were taken at every 4 h of both top insert and bottom reservoir, and total cell occupancy in the bottom reservoir normalized to initial top value was determined. Mean rate (MR) was determined by calculating the slopes of the curve.

### 2.7 Proliferation assay

Cell proliferation was determined by IncuCyte Zoom live cell analysis system from Essen Bioscience. 2000 cells /well were seeded in a 96‐well plate with 10% FBS‐containing media. Cells were incubated in the IncuCyte Zoom incubator at 37 °C with 5% CO_2_. Images were taken at every 4 h, and cell occupancy percentage over time was determined. MTS proliferation assay was conducted by seeding 3000 cells/well in a 96‐well plate following the manufacturer’s protocol (Promega, Madison, WI, USA, cat# G3580).

### 2.8 Co‐immunoprecipitation (co‐IP)

200 µg of cell lysate was incubated with TLK1‐ or GFP‐specific antibodies for 4 h on ice. 50 µL of pre‐equilibrated protein A/G agarose beads (50% slurry) (SCBT, Dallas, TX, USA, cat# sc‐2003) was added and incubated overnight at 4 °C with rotation. Beads were washed three times with cell lysis buffer, and bound proteins were eluted with 1X Laemmli buffer. The eluted sample was further subjected to WB analysis.

### 2.9 Protein purification

Recombinant full‐length his‐tagged TLK1B and his‐tagged MK5 protein were purified according to the published literature [12, 40].

### 2.10 His and GST pulldown assay

Cell lysates were prepared in 1X RIPA lysis buffer (SCBT, Dallas, TX, USA, cat# 24948). 20 µg of His‐tagged TLK1B or GST‐tagged MK5 (Sino Biological, Chesterbrook, PA, USA, cat# 13655‐H20B) was added to 400 µg of cell lysates and incubated on ice for 30 min. 30 µL of equilibrated Ni^2+^‐NTA agarose (50% slurry) (Qiagen, Germantown, MD, USA, cat# 30210) or glutathione agarose (50% slurry) (Thermo Fisher, Waltham, MA, USA, cat# 16100) was added to the mixture and incubated on ice for 30 min. The reactions were spun down, and beads were washed three times with 1X RIPA lysis buffer. Bound proteins were eluted using 1X Laemmli buffer and run into SDS/PAGE gel for WB analysis.

### 2.11 ADP hunter assay

ADP hunter assay was conducted to determine the catalytic activity of laboratory‐purified recombinant MK5 kinase using either HSP27 (Abcam, Cambridge, MA, USA, cat# ab48740) or PRAKtide (KKLRRTLSVA) (Alan Scientific, Gaithersburg, MD, USA), as previously described [12].

### 2.12 *In vitro* kinase assay

Radioactive *in vitro* kinase (IVK) assay was conducted by incubating HSP27 with either TLK1B or MK5 or with both TLK1B and MK5, as previously described [12]. For mass spectrometric (MS) analysis, another IVK assay was conducted by incubating either MK5 or TLK1 with nonradiolabelled ATP. MK5 gel bands were excised and sent to the Kentucky MS facility.

### 2.13 Identification of MK5 phosphorylation by mass spectrometry

Identification of the phosphoresidues of MK5 by TLK1 was conducted at the University of Kentucky Proteomics Core Facility as previously described [12].

### 2.14 Site‐directed mutagenesis (SDM)

Point mutations on MK5 open reading frame in the eGFP‐MK5 plasmid were introduced using a QuikChange Lightning Site‐Directed Mutagenesis Kit (Agilent Technology, Santa Clara, CA, USA, cat# 210519), following the manufacturer’s protocol. The following primers were used to generate the K51E and S354A mutations (only the forward primers are shown): K51E: 5’‐CGGTTTGCACTGGAAATTCTTCTTGATCG‐3’; S354A: 5’‐CTCAAACCCCTGCACGCTGTCAACAACCCCATT‐3’. All mutations were verified by sequencing.

### 2.15 Direct fluorescence microscopy

eGFP‐MK5‐ or eGFP‐MK5+TLK1‐overexpressing HEK 293 cells were seeded in a four‐chamber glass slides (Corning, Glendale, AZ, USA, cat# 354114) at 5000 cells·well^−1^. After 48 h of cells attachment, media were aspirated out, and cells were washed twice with 1X PBS and fixed with 4% formaldehyde solution for 15 min at room temperature. After washing, cells were permeabilized in 0.1% Triton X‐100 in PBS for 15 min. Cells were washed twice with PBS, mounted and coverslipped with VECTASHIELD antifade mounting medium with DAPI (Vector Laboratories, Burlingame, CA, USA, cat# H‐1200). GFP signal distribution was monitored using a Nikon Ti2 fluorescent microscope.

### 2.16 Immunohistochemistry

For TRAMP mouse IHC staining, prostates were harvested, formalin‐fixed, processed and paraffin‐embedded. Prostate TMA was prepared as previously described [10], and its layout is outlined in Table TS3. Tumour tissues were cut in serial 5‐µm‐thin sections, deparaffinized in xylene, rehydrated with decreased concentrations of ethanol and boiled in unmasking solution (10 mm sodium citrate + 1 mm EDTA) for 15 min for antigen retrieval. Cellular peroxidase was quenched with 3% hydrogen peroxide. Afterwards, tissue sections were blocked in 3% BSA for 1 h followed by the incubation of primary antibody against pMK5 Ser354 (1:200 dilutions) overnight at 4 °C. Sections were washed three times and incubated in secondary antibody (Vector Laboratories, Burlingame, CA, USA, cat# PK‐6200) for 1 h at room temperature. After washing, the sections were then incubated in Vectastain ABC reagent (Vector Laboratories, Burlingame, CA, USA, cat# PK‐6200) for 30 min. The sections were washed again and incubated in DAB substrates for 2 min (Vector Laboratories, Burlingame, CA, USA, cat# SK‐4105). Slides were then washed, counterstained with haematoxylin, dehydrated and coverslipped. Imaging and quantification were done by our pathologist, J. King.

### 2.17 Western blotting

Cell were harvested and lysed in 1X RIPA buffer by sonication. Protein concentration was measured using Pierce BCA Protein Assay Kit (Thermo Scientific, Waltham, MA, USA, cat# 23225). Samples were run in SDS/PAGE and transferred to a PVDF membrane. After blocking in 5% nonfat dry milk, the membranes were incubated in primary antibodies overnight at 4 °C followed by the incubation in HRP‐conjugated secondary antibodies for 1 h at room temperature. The blots were incubated in ECL substrates (Thermo Scientific, Waltham, MA, USA, cat# 32106), and imaging was conducted using Bio‐Rad ChemiDoc Imaging System (Bio‐Rad, Hercules, CA, USA, cat# 12003154).

### 2.18 Bioinformatics analysis

Genomic amplification and mRNA expression of both TLK1 and MK5 based on regional lymph node metastases and Gleason scores were generated interrogating The Cancer Genome Atlas Prostate Adenocarcinoma (TCGA‐PRAD) database using cBioPortal (accessed on 7 February 2020) and UALCAN online platform (accessed on 7 February 2020) [45]. TCGA‐PRAD data set can also be downloaded from TCGA data portal (https://tcga‐data.nci.nih.gov/tcga/tcgaHome2.jsp). The Kaplan–Meier survival plot of PCa patients based on high and low TLK1 expression along with different Gleason scores was generated using UALCAN online platform (accessed on 24 November 2021).

### 2.19 Statistical analysis

Statistical calculations were performed using graphpad prism 9 and microsoft excel software. Data quantifications are expressed as mean± standard error of the mean (SEM). Statistical significance was calculated by 2‐tailed Student’s *t*‐test when comparing the mean between two groups, or by one‐way ANOVA followed by Tukey’s *post hoc* analysis when comparing more than two groups. *P*‐values < 0.05 were considered significant.

### 2.20 Ethics approval and consent to participate

Construction of the PCa TMA was made at LSUHSC‐Shreveport following the Institutional Review Board (IRB) approval. The experiments were undertaken with the understanding and written consent of each subject. Furthermore, the identity of each donor was deleted from the construction of the block. The study methodologies conformed to the standards set by the Declaration of Helsinki. Animal studies were approved by our institutional animal care (IACUC) for our previous work published in Ref. [10], and only archival tumour tissue from TRAMP mice [46] was used. No animals were used for the purpose of this work.

## 3 Results

### 3.1 Genetic depletion and pharmacologic inhibition of both MK5 and TLK1 result in reduced cell migration rate

**Fig. 1:**
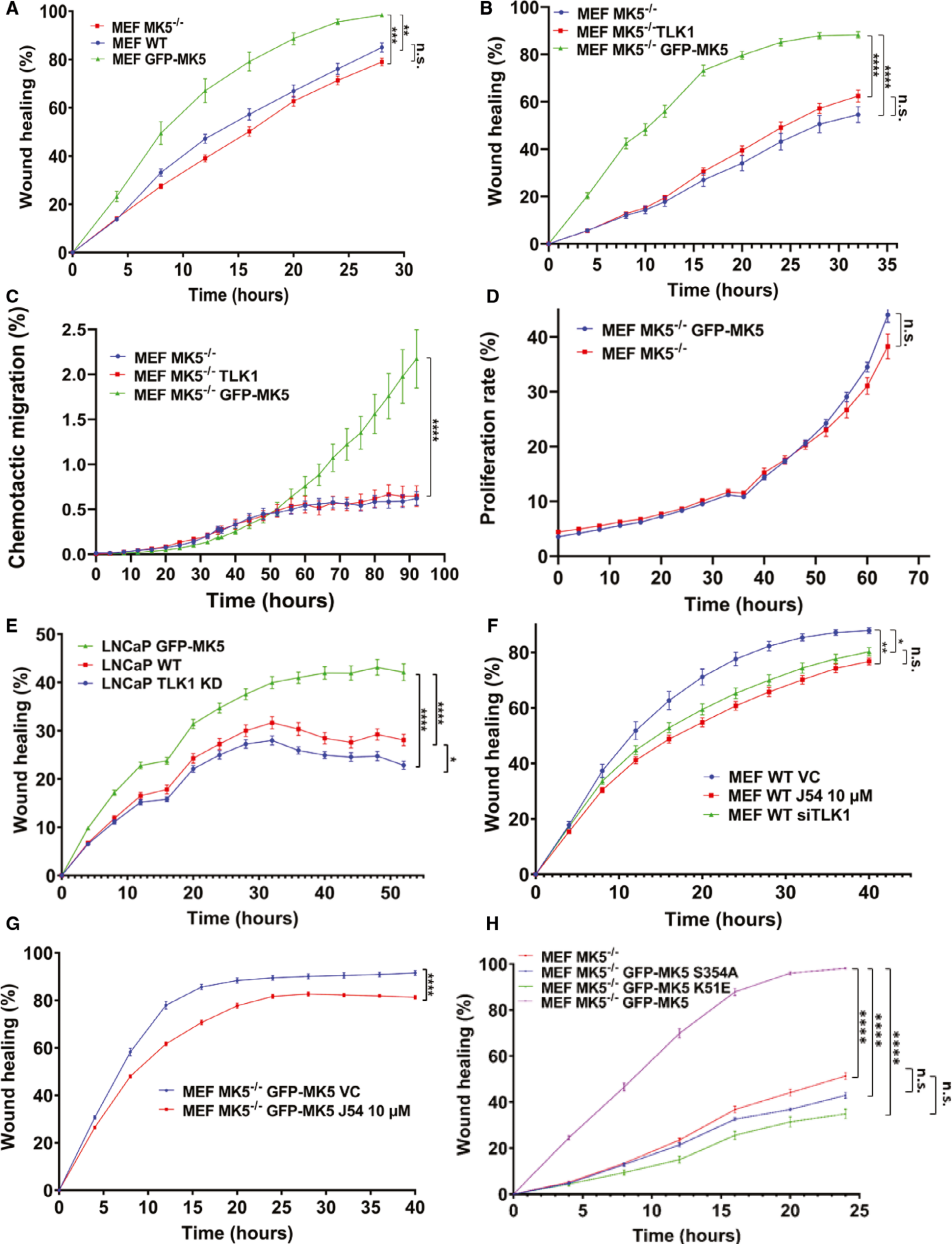
Both MK5 and TLK1 regulate cell migration. A, B, E, F, G and H) Scratch wound repair assay was conducted to determine the 2D migration rate by plotting relative wound density against different time points. (A) Wild‐type MEF (MEF WT), mean rate (MR): 10.97 ± 0.45; MEF MK5^−/−^, MR: 9.40 ± 0.35; and MEF GFP‐MK5, MR: 15.32 ± 1.2. (B) MEF MK5^−/−^, MR: 4.23 ± 0.30; MEF MK5^−/−^ TLK1, MR: 5.19 ± 0.22; and MEF MK5^−/−^ GFP‐MK5, MR: 13.69 ± 0.57. (E) LNCaP WT, MR:3.97 ± 0.10; LNCaP TLK1 kinase dead (KD), MR:3.47 ± 0.11; and LNCaP GFP‐MK5, MR: 5.10 ± 0.16. (F) MEF wild‐type vehicle control (MEF WT VC, MR: 12.18 ± 0.71); MEF WT J54 10 µm, MR: 9.65 ± 0.30; and MEF WT siTLK1, MR: 10.51 ± 0.37. (G) MEF MK5^−/−^ GFP‐MK5 VC, MR: 17.40 ± 0.40; MEF MK5^−/−^ GFP‐MK5 J54 10 µm, MR: 14.39 ± 0.22. (H) MEF MK5^−/−^, MR: 5.62 ± 0.12; MEF MK5^−/−^ GFP‐MK5‐S354A, MR: 5.79 ± 0.55; MEF MK5^−/−^ GFP‐MK5 K51E, MR: 4.96 ± 0.31; and MEF MK5^−/−^ GFP‐MK5, MR: 15.63 ± 0.22. (C) 3D chemotactic trans‐well migration assay among MEF MK5^−/−^, MEF MK5^−/−^ TLK1 and MEF MK5^−/−^ GFP‐MK5 cells at different time points using fibronectin coating. Migration rate was determined by plotting total object phase area against time among MEF MK5^−/−^, MR: 0.04 ± 0.005; MEF MK5^−/−^ TLK1, MR: 0.07 ± 0.014; and MEF MK5^−/−^ GFP‐MK5, MR: 0.16 ± 0.009. (D) Proliferation assay between MEF MK5^−/−^ and MEF MK5^−/−^ GFP‐MK5 cells by plotting confluence percentage over time. MEF MK5^−/−^, MR: 1.93 ± 0.08; MEF MK5^−/−^ GFP‐MK5, MR: 1.97 ± 0.006. 2‐tailed Student’s *t*‐test was used for two group comparison, and one‐way ANOVA followed by Tukey’s *post hoc* analysis was used for multiple group comparison. *=*P* < 0.05, **=*P* < 0.005, ***=*P* < 0.0005, ****=*P* < 0.0001 and n.s. = not significant. Each data point contains 8‐12 replicates. Error bar represents standard error of the mean (SEM).

We have utilized a novel proteomic screening assay from Life Technologies to identify the interactome of TLK1B [47]. TLK1B is a spliced variant of TLK1 that lacks the first 238 amino acids in the N‐terminal domain of TLK1. TLK1B is translationally regulated, shares the identical C‐terminal kinase domain and is believed to have similar substrate specificity with TLK1 [8, 41]. Briefly, biotinylated TLK1B was used to hybridize 9000 human full‐length proteins spotted in duplicates on glass slides, after which fluorophore‐conjugated streptavidin was used to generate the signals from the interactions between TLK1 and arrayed proteins. This assay identified 165 human proteins that interact with TLK1 with high confidence that are involved in various cellular functions such as cell cycle regulation, DNA damage repair, DNA replication and cell motility. MK5 was identified as one of the top interactor proteins (supplementary section of Ref. [47]). Since MK5 promotes cellular motility in both nonmalignant (e.g. PC12 [17], HUVEC [16]) and neoplastic (e.g. HeLa [20]) cells, we tested whether MK5 overexpression or depletion alters the motility rate of mouse embryonic fibroblast (MEF). MK5 was overexpressed in wild‐type (WT) MEF cells using eGFP‐MK5 mammalian construct, and exogenous MK5 level was determined by western blotting (Fig. S1A). We compared the scratch healing rate among MK5^−/−^ MEF‐ [43], WT MEF‐ and MK5‐overexpressing MEF cells by scratch repair assay. Scratch repair appeared to be the fastest in the MK5‐overexpressing MEF and slowest in the MK5^−/−^ MEF cells (Fig. 1A and Fig. S1B).

It is noteworthy that MK5^−/−^ MEF cells were generated in a mixed genetic background (129/Ola X C57BL/6), whereas wild‐type MEF cells were isolated from C57BL/6 mice [43]. To rule out the possibility that the discrepancy in the genetic background of MK5^−/−^ MEF and WT MEF cells can affect their migration rate in scratch wound repair assay, we reconstituted MK5 in the MK5^−/−^ MEF cells (Fig. S2A). In addition, we tested whether TLK1 alone in the absence of MK5 can enhance their migration rate, by stably overexpressing TLK1 in the MK5^−/−^ MEF Cells (Fig. S2B). When we compared the wound healing rates among these three cell lines, namely, MK5‐rescued MEF, MK5^−/−^ MEF and TLK1‐overexpressing MK5^−/−^ MEF cells in a scratch repair assay, we observed that while MK5‐rescued cells migrated significantly faster, there was no significant difference in the migration rate between TLK1‐overexpressing MK5^−/−^ MEF and MK5^−/−^ MEF cells (Fig. 1B and Fig. S2C). The latter results indicate that without MK5, TLK1 cannot exert its function in motility promotion. To confirm our observations from the scratch assay, we utilized the 3D chemotactic trans‐well migration assay in the IncuCyte machine using three different extracellular matrices (ECMs). We observed that in fibronectin, MK5‐rescued cells migrated significantly faster and no difference in the migration rate of TLK1‐overexpressing MK5^−/−^ vs. MK5^−/−^ MEF cells (Fig. 1C and Fig. S3). Similar trends in migration were also observed using Matrigel and collagen I (Fig. S4 and Fig. S5). To examine whether reconstitution of MK5 increases the proliferation rate of the cells, which might contribute to enhanced migration rate that we observed in the scratch and chemotactic migration assays, we conducted a proliferation assay between MEF MK5^−/−^ and MEF MK5^−/−^ GFP‐MK5 cells. MK5 reconstitution did not significantly increase the proliferation rate compared with MK5^−/−^ MEF cells (Fig. 1D and Fig. S6). We conducted another scratch experiment with LNCaP cells, an androgen‐dependent PCa cell line. LNCaP cells were transfected with either eGFP‐MK5 or TLK1 kinase‐dead (KD) dominant negative mammalian construct (Fig. S7A) [41]. We observed significantly faster wound healing in the MK5‐overexpressing LNCaP cells compared with the TLK1 KD and WT LNCaP cells (Fig. 1E and Fig. S7B). Overall, these findings suggest MK5 as a promotility factor; in addition, TLK1 alone in the absence of MK5 cannot increase the migration rate of the cells.

Emerging evidence suggests that TLK2 can promote cancer invasiveness by enhancing the migration and invasion of breast cancer and glioblastoma cell. Since TLK1 and TLK2 share high homology in the amino acid sequence and are believed to have partly redundant functions, we hypothesize that TLK1 can also regulate cellular motility. To examine whether TLK1 acts a potential factor to regulate cell motility, we took both genetic and pharmacologic approaches. Both siRNA‐mediated knockdown and pharmacologic inhibition of TLK1 using small molecule inhibitor (J54) resulted in the reduced scratch healing rate in WT MEF cells compared with the control cells (Fig. S8A, Fig. 1F and Fig. S8B). J54 is a potent second‐generation phenothiazine derivative specific for TLK1 [44]. Scratch healing rate of TLK1‐depleted and TLK1‐inhibited WT MEF cells was similar, and there is no statistical difference between these two experimental groups. Therefore, we opted to use J54 for our next experiments. J54 treatment also significantly reduces the wound healing rate of MK5‐rescued MK5^−/−^ MEF cells compared with the vehicle (DMSO)‐treated MK5‐rescued cells without affecting proliferation or viability (Fig. 1G, Fig. S8C and Fig. S9). These findings establish both TLK1 and MK5 as promotility factors, and we hypothesize that MK5 may function as a downstream effector of TLK1 to promote cellular motility. Unexpectedly, TLK1 inhibition by J54 also resulted in reduced wound closure in MK5^−/−^ MEF cells, which suggest that TLK1 may promote cellular migration through additional downstream effectors even in the absence of MK5 (Fig. S10A and Fig. S10B). We also expressed two mutant variants of eGFP‐MK5: S354A (a target phosphorylation by TLK1—see below) and K51E kinase‐dead variant [42] in MK5^−/−^ MEF cells. Expression of neither protein could restore motility to MK5^−/−^ MEF cells (Fig. 1H, Fig. S11A,B).

### 3.2 TLK1 interacts and phosphorylates MK5 both *in vitro* and in cultured cells and increases its catalytic activity

**Fig. 2:**
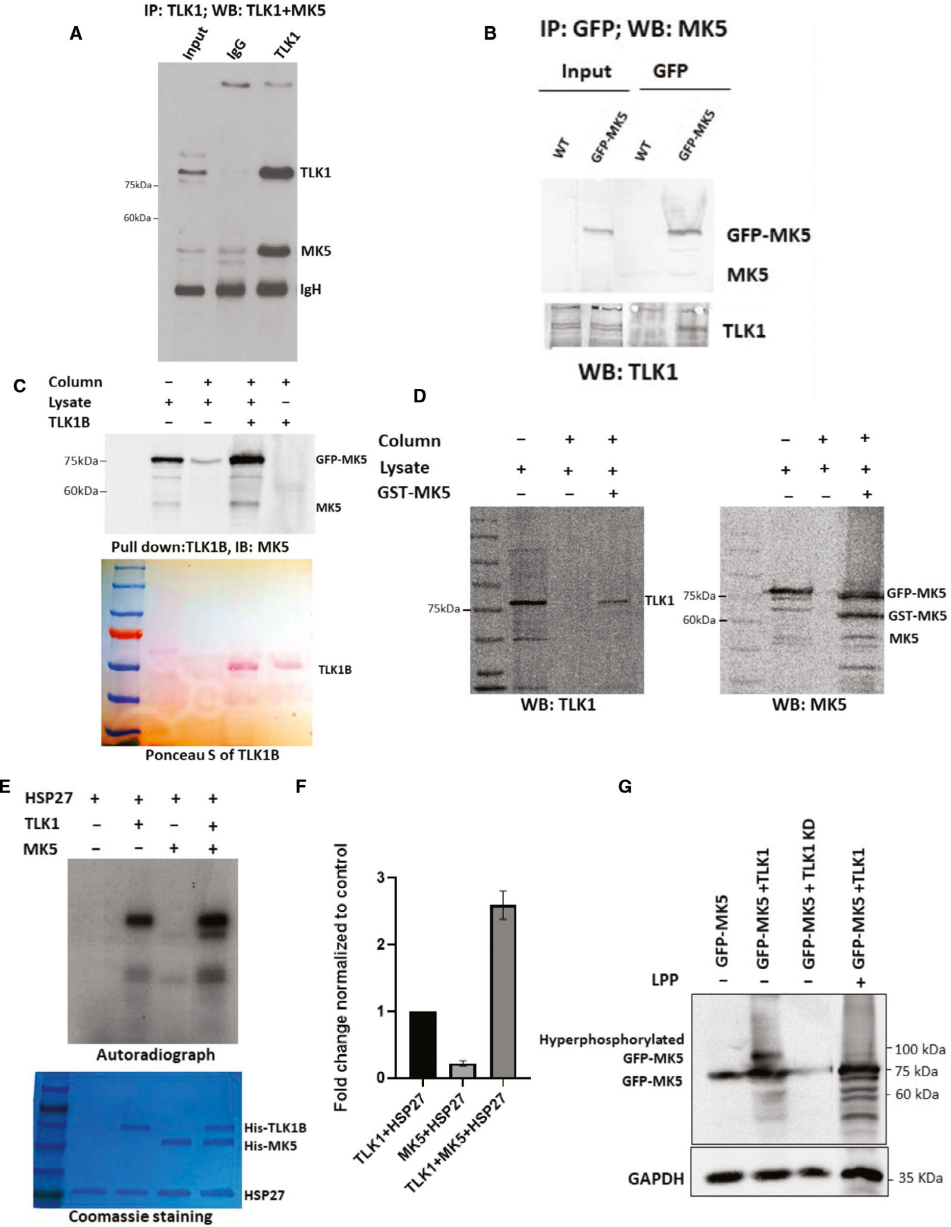
TLK1 interacts and phosphorylates MK5 both *in vitro* and in cultured cells. (A) Co‐immunoprecipitation of endogenously expressed MK5 with TLK1 antiserum from LNCaP cells and blotted sequentially for TLK1 and MK5. (B) GFP‐MK5 was overexpressed in HEK 293 cells. MK5 was immunoprecipitated using GFP‐specific antibody and blotted for both MK5 (upper panel) and TLK1 (lower panel). (C) His pulldown assay using purified his‐tagged TLK1B incubated with GFP‐MK5‐overexpressing HEK 293 cell lysate. Upper panel, WB showing both GFP‐MK5 and endogenous MK5 level. Lower panel, Ponceau S image showing TLK1B band. (D) GST pulldown assay using purified GST‐tagged MK5 incubated with GFP‐MK5 and TLK1 co‐expressing HEK 293 cell lysates. Left panel, WB detection of TLK1. Right panel, WB detection of both GFP‐MK5 and GST‐MK5. (E) An *in vitro* kinase (IVK) assay using recombinant his‐tagged HSP27 incubated with either his‐tagged TLK1B or his‐tagged MK5 or altogether. [ɣ‐^32^P] ATP was used as a radioactive source. Top panel, an autoradiograph showing the intensity of the exposed bands of the corresponding protein. Bottom panel, Coomassie‐stained gel showing equal amount of protein loading. (F) Relative densitometry of the fold change of HSP27 phosphorylation. One‐way ANOVA followed by Tukey’s *post hoc* analysis was used. Error bar represents standard error of the mean (SEM). (G) HEK 293 cells transfected with either GFP‐MK5 alone, or GFP‐MK5+TLK1, or GFP‐MK5+TLK1 KD. Lambda protein phosphatase (LPP) treatment was done in the 4th lane. WB showing hyperphosphorylated GFP‐MK5 band in the 2nd lane, which is reduced by LPP treatment (4th lane). GAPDH was used as a loading control. Each figure is representative of *n* = 3 experiments.

Results obtained from our previously conducted protoarray assay demonstrated that TLK1B interacts with MK5 *in vitro* [47]. To test whether TLK1 interacts with MK5 in cultured cells, we conducted co‐immunoprecipitation (co‐IP) experiments targeting both endogenously and exogenously expressed proteins. Immunoprecipitations of endogenous TLK1 using TLK1‐specific antibodies brought down endogenous MK5 in LNCaP cells (Fig. 2A). We overexpressed MK5 in HEK 293 cells using eGFP‐MK5 mammalian construct and immunoprecipitated MK5 using GFP‐specific antibody, which also precipitated TLK1 (Fig. 2B). These data suggest that TLK1 and MK5 form a complex in cells. We confirmed this observation performing His and GST pulldown experiments independently. Incubation of purified recombinant his‐tagged TLK1B with MK5‐overexpressing HEK cell lysates precipitated both endogenous MK5 and exogenously expressed GFP‐MK5, as determined by WB (Fig. 2C). Similarly, incubation of purified recombinant GST‐tagged MK5 with TLK1 and MK5 coexpressing cell lysate precipitated both TLK1 (Fig. 2D, left panel) and GFP‐MK5 (Fig. 2D, right panel). Together, these results indicate that TLK1 interacts directly with MK5. The observation that endogenous MK5 was also pulled down in both co‐IP (Fig. 2B) and GST pulldown assay (Fig. 2D, right panel) suggests that endogenous MK5 may dimerize with exogenously expressed GFP‐MK5 and/or GST‐MK5.

We examined whether TLK1 possesses the ability to phosphorylate MK5 *in vitro* and in cultured cells. We first purified recombinant his‐tagged TLK1B and recombinant his‐tagged MK5 following the previously published protocol (Fig. S12A,B) [12, 40]. We confirmed the catalytic activity of MK5 using MK5‐specific protein and peptide substrate, HSP27 (Fig. S12C) and PRAKtide (Fig. S12D), respectively, by ADP hunter assay. Incubation of purified recombinant his‐tagged TLK1B with both purified recombinant his‐tagged MK5 and HSP27 in an IVK assay synergistically increases the phosphorylation level of MK5 and intrinsic catalytic activity of MK5 towards its known substrate, HSP27 (Fig. 2E,F). To determine whether TLK1 can phosphorylate MK5 in cultured cells, we transfected HEK 293 cells either with GFP‐MK5 alone or cotransfected the cells either with GFP‐MK5 +TLK1 or with GFP‐MK5+TLK1 kinase‐dead (KD) expression construct. WB analysis revealed a hyperphosphorylated form of GFP‐MK5 only in MK5+ TLK1‐coexpressed cell lysate that migrated slower than the main form of GFP‐MK5 (Fig. 2G). Lambda protein phosphatase (LPP) treatment reduces this slower migrating band, which suggests that TLK1 can phosphorylate MK5 in cultured cells. We also conducted another IVK assay by incubating both recombinant His‐TLK1B and GST‐MK5 in the presence of nonradioactive cold ATP. The reactions were run in an SDS/PAGE gel, and the corresponding MK5 bands were excised for mass spectrometric analysis.

### 3.3 TLK1 phosphorylates MK5 *in vitro* on three unique residues, which are mapped to the functional domains of MK5

To determine the phosphopeptides of MK5 by TLK1, MK5 bands were digested by trypsin and subjected to shot‐gun proteomic analysis using an LTQ‐Orbitrap mass spectrometer. MS data sets are searched with MASCOT against a custom database containing human MAPKAPK5. The summary of the analysis is as follows: (a) MAP kinase‐activated protein kinase 5 (MAPKAPK5 or MK5) was detected with high protein score (4686‐ 8482) and good peptides coverage (70.40‐80.55%) in both samples. (b) Phosphorylated sites were detected in both samples. After comparing MK5 mock and MK5+TLK1B samples, a few unique phosphorylation sites were detected in MK5+TLK1B sample: S160, S348, S354 and S386 (Fig. S13 and Table S1). The constitutive phosphorylation sites present in both MK5 mock and MK5+TLK1B samples can be explained as the autophosphorylation sites of MK5 (Fig. S14). (c) S348 and S354 are within the same peptide with single phosphorylation event. After comparing the spectra, S354 was determined to be the more likely phosphorylation site. (d) Thus, we conclude that there are three unique phosphorylation sites in MK5+TLK1B sample: S160, S354 and S386. We mapped these serine residues to the domains of MK5 and identified that they are located in the kinase domain (in the activation loop near the residue Thr182, which needs to be phosphorylated for the catalytic activation of MK5 [48, 49]), in the nuclear localization signal (NLS) domain and in the ERK3 binding domain (rev. in [37]) (Fig. S15).

### 3.4 Antiandrogen treatment increases pMK5 Ser354 level and can be inhibited by small molecule inhibitor specific to TLK1

**Fig. 3:**
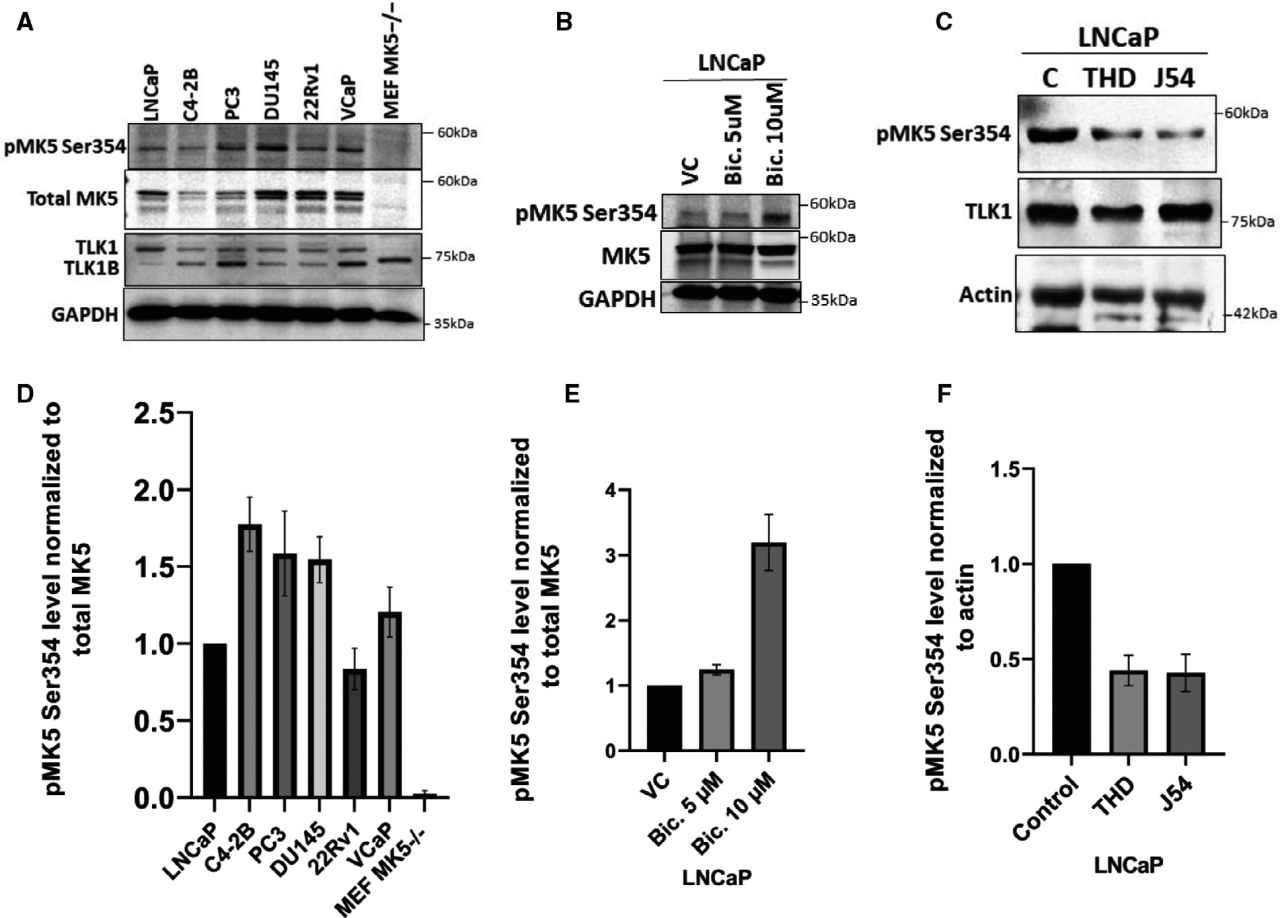
Antiandrogen treatment increases pMK5 Ser354, and TLK1 inhibition reduces it. (A) pMK5‐S354, total MK5 and TLK1/1B level in different androgen‐dependent and androgen‐independent PCa cell lines determined by WB. GAPDH was used as a loading control. (B) pMK5 Ser354 level in LNCaP cells after two different concentrations of bicalutamide treatment compared with the vehicle control (VC) determined by WB. (C) pMK5‐S354 and TLK1 level in LNCaP cells after treatment with two different TLK1 inhibitors (THD and J54) at 10 µm concentration. (D) Densitometric quantification of pMK5‐S354 level normalized to total MK5 in different PCA cell lines. (E) Densitometric quantification of pMK5‐S354 level normalized to total MK5 upon bicalutamide treatment. (F) Densitometric quantification of pMK5‐S354 level normalized to loading control upon two different TLK1 inhibitor treatments. Each figure is representative of three replicates. One‐way ANOVA followed by Tukey’s *post hoc* analysis was used for multiple group comparison. Error bar represents standard error of the mean (SEM).

Antiserum raised against one of the phosphoresidue of MK5 (Ser354), one of the sites that we identified as being target of TLK1, has recently become commercially available, and we tested it to determine whether this specific MK5 phosphoprotein is present in several androgen‐dependent and androgen‐independent PCa cell lines (Fig. 3A and D). MK5^−/−^ MEF cells were used as a negative control. We also detected TLK1 in those PCa cell lines (Fig. 3A). Western blotting analysis revealed the presence of pMK5‐S354 level relative to the total MK5 in all PCa cell lines tested, while no pMK5‐S354 was detected in MK5^−/−^ MEF cells. We also previously reported that treatment of LNCaP and other androgen‐sensitive PCa cells with bicalutamide results in increased expression of TLK1B [9, 10]. We now show that antiandrogen treatment also results in a corresponding dose‐dependent increase in pMK5 (S354) (Fig. 3B and E), suggesting that TLK1/1B may be the kinase responsible for this phosphorylation. Moreover, we treated LNCaP cells with two specific small molecule inhibitors of TLK1 (THD or J54), which resulted in the reduction of pMK5‐S354 level compared with the DMSO‐treated control LNCaP cells, which further supports TLK1 role in MK5 Ser354 phosphorylation (Fig. 3C and F).

### 3.5 pMK5 Ser354 is elevated in PCa progression of TRAMP mice and in patients with high‐grade metastatic prostate cancer

**Fig. 4:**
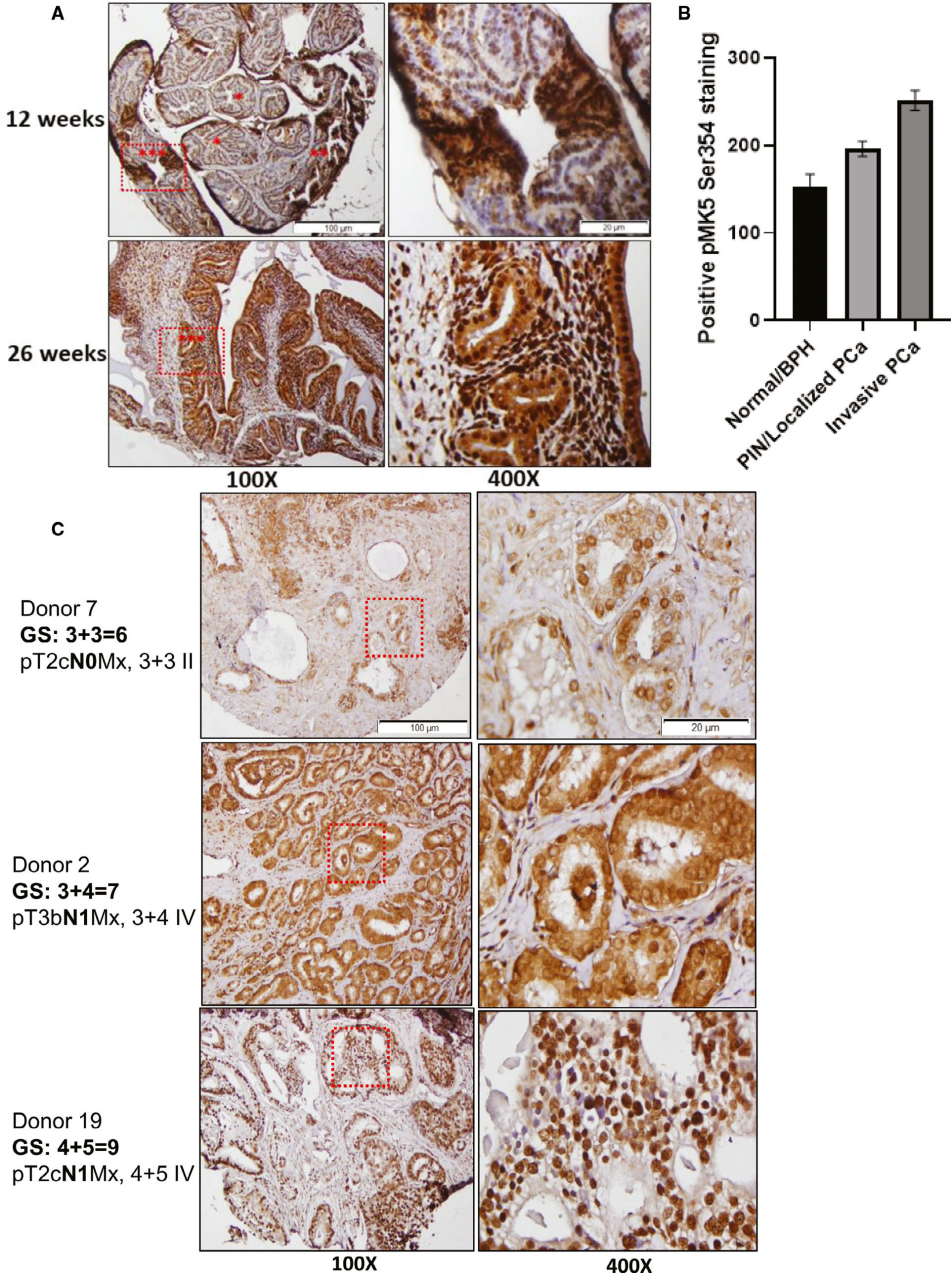
IHC staining revealed elevated pMK5 Ser354 level in PCa progression of TRAMP mice and in patients with high‐grade metastatic prostate. (A) Top panel, pMK5 Ser354 staining in a mixture of normal tissue/BPH, PIN/localized PCa and invasive PCa regions in 12‐week‐old mice. Bottom panel, pMK5 Ser354 staining in the invasive PCa lesions of 26‐week‐old mice that have spread throughout the gland. *= normal tissue/BPH, **= PIN/localized PCa and ***= invasive PCa. (B) ImageJ analysis of the positive staining of pMK5 Ser354 in different regions of TRAMP prostate tumour. Each figure is representative of three different TRAMP prostate tumours. One‐way ANOVA followed by Tukey’s *post hoc* analysis was used. Error bar represents standard error of the mean (SEM). (C) pMK5 Ser354 staining in TMA samples of Gleason scores 6, 7 and 9. Black dashed boxes represent the magnified regions. Scale bar: 100 µm (100×) and 20 µm (400×).

We wanted to study whether the presence of pMK5 presented with a pattern of progressive intensity during progression to invasive PCa in the TRAMP model, given that we have already reported the expression of TLK1B increases greatly from 12 to 30 weeks of age, particularly after castration [10]. At 12 weeks, TRAMP mice present feature of early carcinogenic progression of the prostate, with a mix of hyperproliferative lesions such as prostatic intraepithelial neoplasia (PIN), to well‐differentiated adenocarcinomas, and in some case some areas of invasiveness with breakdown of the basement membrane. These features are remarkably well represented in Fig. 4A (top panels), which clearly shows that the areas of PIN, confined PCa and locally invasive PCa are stained progressively darker upon pMK5‐IHC, compared with normal acini (Fig. 4A and B). At 26 weeks, the PCa has progressed to fully invasive, and the cancer cells are spread thought the glands (Fig. 4A, bottom panels).

We interrogated our institutional PCa TMA (with African American prevalence) to establish possible clinicopathologic correlations based on the Gleason score/s (GS), regional lymph node metastasis and the levels of pMK5 Ser354 (Table S2). pMK5 Ser354 staining intensity was progressively higher in tumour samples with increasing Gleason scores and lymph nodes metastases. For instance, our representative IHC images revealed higher positive staining of pMK5‐S354 of several GS 8‐9 tumours compared with GS 6 (Fig. 4C). Similar increase was also observed in the tumours with 1‐3 lymph node metastasis (N1). A very notable feature was that regardless of GS (the key token of pathology determination to date), the nuclear‐to‐cytoplasm ratio of pMK5 was decreased in several samples, including some N1 of not the highest GS grade (Table S3). MK5 is generally localized to the nuclei, but it shuttles to the cytoplasm upon MAPK activation [42, 50], where it is believed to regulate actin cytoskeletal reorganization, although the role of stress‐responsive p38‐MAPK in activating MK5 and its redistribution is debated [42, 43]. We have thus investigated the possible role of TLK1‐overexpressing cells in the cellular sublocalization of eGFP‐MK5 and found that at steady state and in the absence of cellular stress, the distribution of eGFP‐MK5 was strongly shifted to the cytoplasm in contrast to the nucleus of cells with normal level TLK1 (Fig. S16). While our TMA does not report on patients’ outcome, it would be intriguing to study this in the future to establish whether the pMK5 nuclear/cytoplasm ratio can be used as a prognostic indicator. Generally, tumours with GS≤ 6 are considered as low‐risk tumours with well‐differentiated PCa cells, GS=7 is considered as moderate risk, and GS≥ 8 is considered as high‐risk tumours with poorly differentiated PCa cells that have higher migratory and invasive potentials. Higher phosphorylation of MK5 may suggest TLK1‐MK5 signalling as a key driver of metastasis in PCa subjects.

### 3.6 Pharmacologic inhibition of MK5 strongly reduces wound healing of both androgen‐dependent and androgen‐independent PCa cells

**Fig. 5:**
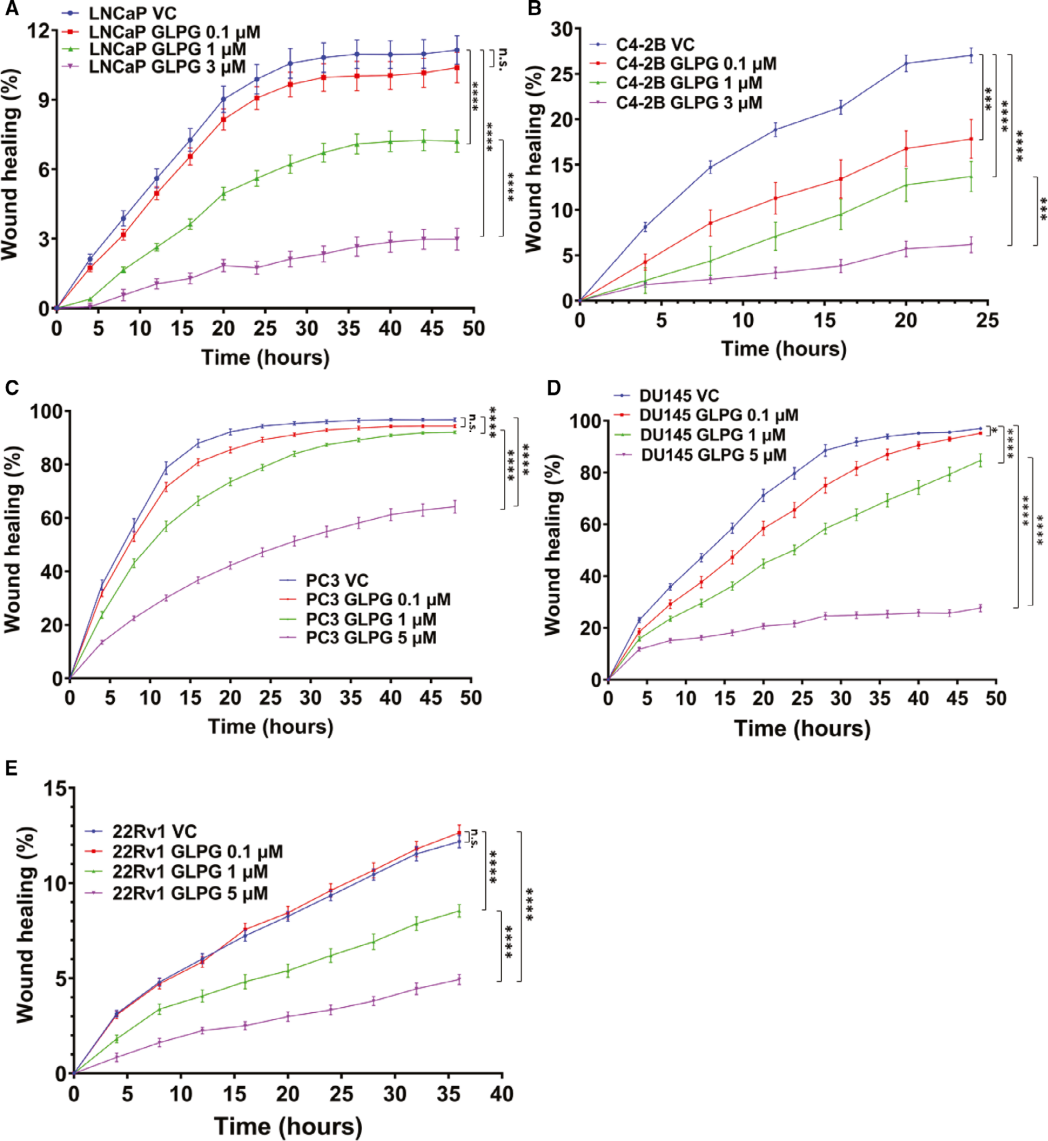
MK5 inhibition reduces wound healing in PCa cell lines. Scratch wound repair assay was conducted to determine the 2D migration rate by plotting relative wound density against different time points. (A) LNCaP vehicle control (VC), MR: 1.08 ± 0.05; LNCaP GLPG 0.1 µm, MR: 1.00 ± 0.05; LNCaP GLPG 1 µm, MR: 0.60 ± 0.04 cells; and LNCaP GLPG 3 µm, MR:0.21 ± 0.04. (B) C4‐2B VC, MR: 4.47 ± 0.14; C4‐2B GLPG 0.1 µm, MR: 2.72 ± 0.34; C4‐2B GLPG 1 µm, MR: 1.87 ± 0.31; and C4‐2B GLPG 3 µm, MR: 0.83 ± 0.14. (C) PC3 VC, MR: 9.98 ± 0.08; PC3 GLPG 0.1 µm, MR:9.65 ± 0.08; PC3 GLPG 1 µm, MR: 8.98 ± 0.11; and PC3 GLPG 5 µm, MR:5.68 ± 0.21. (D) DU145 VC, MR: 9.07 ± 0.13; DU145 GLPG 0.1 µm, MR: 8.26 ± 0.23; DU145 GLPG 1 µm, MR: 7.00 ± 0.25; and DU145 GLPG 5 µm, MR: 2.60 ± 0.12. (E) 22Rv1 VC, MR: 1.32 ± 0.05; 22Rv1 GLPG 0.1 µm, MR: 1.34 ± 0.05; 22Rv1 GLPG 1 µm, MR: 0.85 ± 0.05; and 22Rv1 GLPG 5 µm, MR: 0.47 ± 0.03. *=*P* < 0.05, ***= *P* < 0.0005, ****=*P* < 0.0001 and n.s. = not significant. Each data point contains 8‐12 technical replicates and *n* = 3 independent experiments. One‐way ANOVA followed by Tukey’s *post hoc* analysis was used for multiple group comparison. Error bar represents standard error of the mean (SEM).

To establish that MK5 is indeed important for cell motility, we tested a panel of PCa cell lines, including LNCaP, C4‐2B, PC3, DU145 and 22RV1 with the specific MK5 inhibitor GLPG0259 (Fig. 5 and Fig. S17) using the scratch repair assay via the IncuCyte. In all cell lines tested, GLPG resulted in a strong immobilization effect with no apparent toxicity in terms of loss of viable cells. This effect was noticeable within few hours after the addition of the drug, with already maximal effect at 5 μm, which is close to the established IC_50_ for GLPG0259 [51, 52]. We should emphasize that GLPG0259, at the concentrations used in these assays (which are similar to those reported in human studies [51]), did not affect viability and growth. While we have not yet tested GLPG0259 on a cell line such as PC3 in a xenograft model of metastasis, such work was reported recently with very exciting results of metastasis suppression in xenografts of B16 and MDA‐MB‐231 cells [53].

### 3.7 Bioinformatic analysis revealed genomic amplification and upregulation of both TLK1 and MK5 in metastatic tumours

**Fig. 6:**
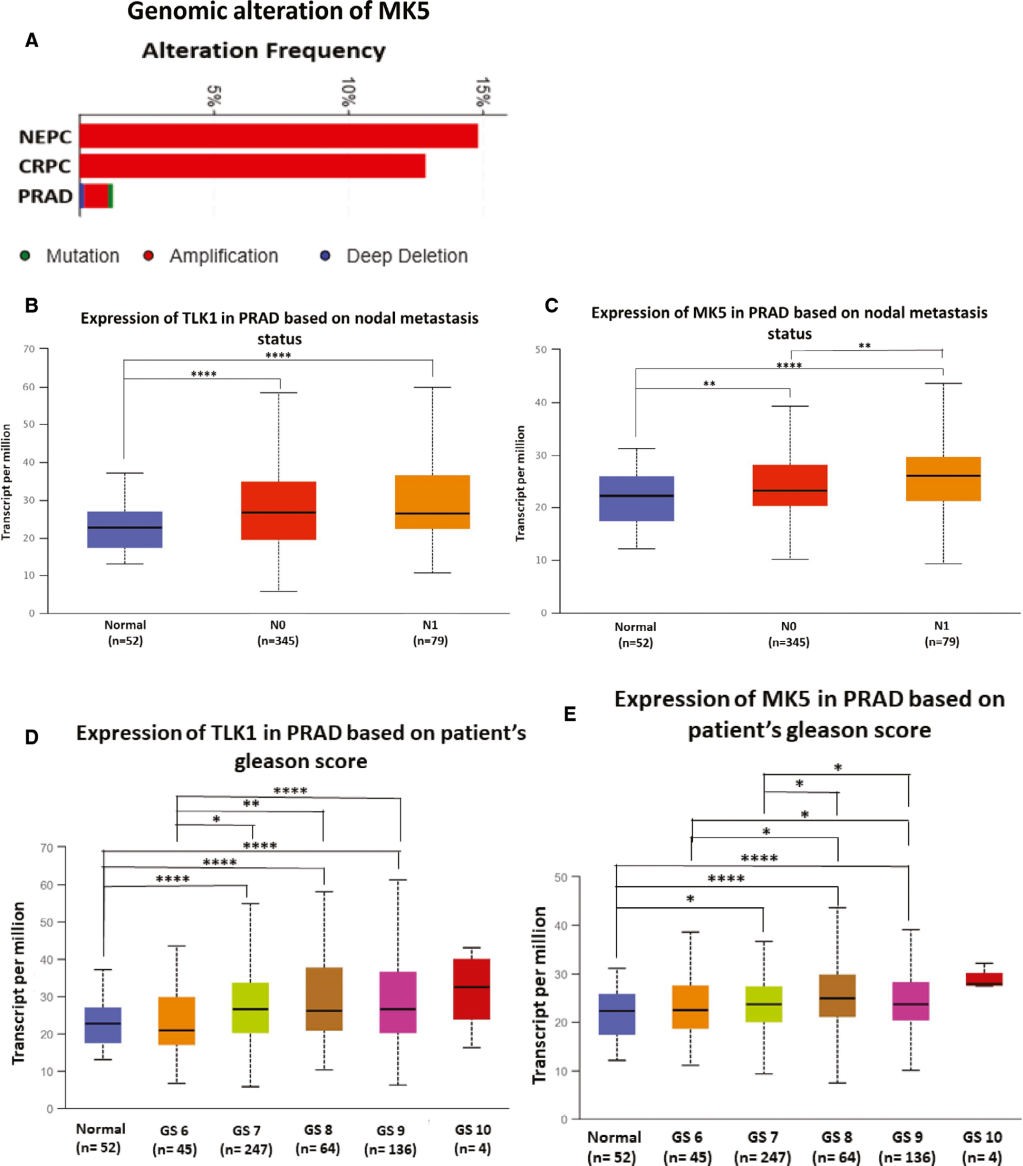
Copy‐number increase and upregulation of TLK1 and MK5 in metastatic PCa. Bioinformatic analysis of PCa data sets to determine: (A) genomic alteration of MK5; (B) mRNA expression of TLK1 based on nodal metastasis; (C) mRNA expression of MK5 based on nodal metastasis; (D) mRNA expression of TLK1 based on tumour Gleason score; (E) mRNA expression of MK5 based on tumour Gleason score. CRPC= castration‐resistant prostate cancer, PRAD = prostate adenocarcinoma, NEPC = neuroendocrine prostate cancer. Box‐and‐whisker plots represent interquartile range (IQR) including minimum, 25th percentile, median, 75th percentile and maximum values. N0 = no regional lymph node metastasis and N1 = metastases in 1 to 3 axillary lymph nodes. GS = Gleason score. *=*P* < 0.05, **=*P* < 0.005 and ****=*P* < 0.0001.

Previous studies reported TLK1 as one of the major drivers of CRPC progression after ADT [9, 10, 12]. Androgen ablation also results in the activation of some MAPK pathways, which will further increase tumour aggressiveness. We interrogated publicly available databases to determine TLK1 and MK5 status in actual PCa patients with advanced tumours. Analysis of TCGA, SU2C, Broad/Cornell and other PCa data sets revealed genomic copy‐number increase of MK5 in CRPC patients compared with localized tumours (Fig. 6A). We analysed the mRNA expression of both TLK1 and MK5 of PCa tumours with different nodal metastatic status using the UALCAN online bioinformatic tool. Tumours with 1‐3 regional nodal metastatic lesions (N1) showed slightly higher expression of TLK1, but significant upregulation of MK5 compared to tumours with no nodal metastatic lesion (N0) (Fig. 6B and C) was observed. Similarly, consistent upregulation of both TLK1 and MK5 was observed in tumours with increasing Gleason scores (Fig. 6D and E), although our driving hypothesis is that it is the activity of MK5 (via upregulation by TLK1) that is really more significant. Importantly, the TLK1 gene was identified by coexpression analysis using WGCNA as a key driver of PCa, highly enriched among candidate genes collected from expression quantitative trait loci (eQTL), somatic copy‐number alterations (SCNA) and prognostic analyses [54]. Further analysis of TLK1 stratified as high or low expression indicated that it is a predictor of poor outcome, with earlier death time, when the Gleason score is lower and thus deemed as more favourable (Fig. S18).

## 4 Discussion

Prostate cancer (PCa) is a slow‐progressing disease where the mortality rate peaks with the metastasis of cancer cells to other vital organs. While organ‐confined PCa is more clinically manageable and has a nearly 100% five‐year survival rate, PCa metastasis drastically decreases the survival of the patients. The median survival rate of the prostate cancer patients with liver, lung, bone and lymph node metastasis is approximately 14, 19, 21 and 32 months, respectively [55]. Failure of ADT leads to more aggressive malignancy and drives metastatic progression of the PCa. However, the molecular mechanism of this metastatic dissemination is not fully understood yet. Motility and invasion are two essential cellular processes for cancer cells to get detached from the primary tumour site and disseminated throughout the body. Motility and invasion are regulated by the concerted action of several factors, coordination of multiple signal transduction pathways, gene expression, cytoskeletal changes and remodelling of surrounding extracellular matrices. These allow the cells to invade and migrate through new tissues.

Our discovery of TLK1B‐MK5 interaction through protoarray assay prompted us to investigate the role of TLK1 and MK5 in motility in both nonmalignant and neoplastic cell lines as TLK1 paralog, TLK2 and another kinase, MK5, were reported to increase cellular motility and invasion [13, 14, 16, 18, 20]. Both genetic depletion and pharmacologic inhibition of MK5 and TLK1 independently resulted in significantly reduced wound healing, whereas overexpression/rescue of MK5 enhances the wound closure rate (Figs. 1 and 5). However, overexpression of TLK1 alone in MK5‐depleted cells did not promote the migration, which suggests TLK1 must exert its promotility function primarily through MK5 (Fig. 1B,C). We further confirmed TLK1‐MK5 interaction by co‐IP, His and GST pulldown assay and elucidated that TLK1 can phosphorylate MK5 in three serine residues (S160, S354 and S386), which are located on some functional domains of MK5 (Figs. 2 and S13‐S15). Thus, MK5 is a direct substrate of TLK1, which increases its intrinsic kinase activity *in vitro* (Fig. 2E and 2G). We reported the presence of TLK1 and MK5 phosphoprotein (pMK5‐S354) in all major androgen‐dependent and androgen‐independent PCa cell lines and demonstrated that antiandrogen (bicalutamide) treatment increases the pMK5‐S354 level in a dose‐dependent fashion (Fig. 3A,B,D and E). Pharmacologic inhibition of TLK1 reduces this pMK5 level, which suggests TLK1 as an authentic pMK5‐S354 kinase (Fig. 3C and 3F). The importance of S354 phosphorylation for the activation of MK5 was then demonstrated by the lack of motility rescue of MK5^−/−^ MEF cells complemented with a MK5‐S354A mutant, which was no better than the K51E kinase‐dead variant (Fig. 1H and Fig. S11). In this respect, one could argue that TLK1 appears to play a critical role in the activation of MK5 at least for some of its functions. In fact, overexpressing TLK1 resulted in the relocalization of GFP‐MK5 to cytoplasm in the absence of a cellular stress (e.g. osmotic) that might involve the activation of p38‐MAPK [42], or by invoking the action of PKA (see below).

In PCa, TLK1/1B‐MK5 signalling axis might be crucial as the ADT‐related stress increases TLK1/1B level and activates compensatory MAPK signalling, which may result in the activation and stabilization of MK5 [9, 56]. Through our bioinformatics study using TCGA and other publicly available data sets, we also observed that both TLK1 and MK5 are upregulated in metastatic and advanced PCa (Fig. 6). Cellular abundance of these kinases may increase their interaction frequencies and start a novel signalling transduction pathway to promote cellular migration. When the tumour grows sufficiently large, cells may shed from the primary tumour site and find a secondary location to avoid hypoxic stress. We propose that TLK1‐MK5 signalling aids this process by increasing the motility and invasion of the cells. Interrogation of a PCa TMA and IHC of TRAMP mice of different stages of PCa progression leading to metastatic spread is supportive of the notion that pMK5‐S354 is potential marker of disease progression and in particular of invasive PCa (Fig. 4). Notably to date, pathologists mainly use H&E staining in the attempt to determine PCa features that predict worse outcome. However, a relatively mild GS is often no predictor of better outcome, particularly if accompanied by the known presence of nodal involvement (N1). A reliable IHC marker, with an established cellular function, that better predicts the intrinsic invasive potential of the PCa cells would be a significant addition to available tools for pathology determinations.

Mechanistically, cellular motility is orchestrated by the formation of protruding bodies (lamellipodia, filopodia, invadopodia), which are rich in submembranous actin filaments. MK5 can regulate actin cytoskeletal reorganization through its bona fide substrate HSP27. Nonphosphorylated HSP27 functions as a capping protein to inhibit F‐actin polymerization and thus inhibits stress fibre formation [57, 58, 59, 60, 61]. Upon external stress‐related stimuli, activated MK5 can phosphorylate HSP27 on Ser15, Ser78 and Ser82 residues, which hinders its capping activity and allows monomeric actin to grow into filamentous actin [17, 18, 20, 48, 62, 63]. Ugo Moens group demonstrated that catalytic subunit of PKA can interact and phosphorylate MK5 on Ser115 residue, which induces its nuclear export and F‐actin polymerization [17, 18, 64]. Similarly, TLK1 interaction and phosphorylation of MK5 may also promote cellular motility by actin remodelling mediated through HSP27 phosphorylation. In fact, our IVK analysis shows that TLK1 increases MK5 catalytic activity towards HSP27 phosphorylation (Fig. 2E and 2F).

Another potential mechanism could be focal adhesion complex modifications by TLK1‐MK5 signalling that promotes cell motility. Since both TLKs and MK5 are known to form complexes with FAK [16, 22], Src [13, 14, 22] and paxillin [22], it is likely that these complexes depend on TLK1‐MK5 interaction. TLK2 is reported to complex with Src and activate EGFR/Src/FAK signalling pathway to promote the migration and invasion in breast adenocarcinoma and glioblastoma cells [13, 14]. Yoshizuka et al. demonstrated that MK5 increases motility in endothelial cells by phosphorylating FAK on Tyr397 residue and localizes it to focal adhesions in advanced skin carcinogenesis [16]. Additionally, another group reported that recombinant MK5 can phosphorylate FAK, Src and paxillin in IVK assays and form complexes with FAK and Src and that endogenous MK5 localizes to focal adhesions [22]. We suggest that TLK1 interacts with MK5 and recruits it to focal adhesions where both TLK1 and MK5 may form complex with either FAK or Src or paxillin, phosphorylate and activate them and transduce downstream signals. FAK activation by TLK1‐MK5 axis can regulate the affinity and avidity of integrins towards ECM and initiate a feedforward loop of MK5 activation through Rac1 > PAK > ERK3/4 > MK5 signalling [65, 66, 67, 68].

MK5 can be activated by both typical and atypical MAPKs, p38 [48] and ERK3/4 [69, 70, 71], respectively. MK5 full activation needs the phosphorylation of Thr182 residue, which is located in its activation loop [48, 70]. One of the phosphorylation of MK5 by TLK1 is mapped to Ser160, and this site is located on the catalytic domain near to the activation loop of MK5. This phosphorylation may cause a conformational change of MK5 and increase its affinity towards its substrates. On the contrary, TLK1 phosphorylation of MK5 may also enhance nucleocytoplasmic localization of MK5, while MK5 is predominantly nuclear in the resting cells. This is, in agreement with the findings of others that phosphorylation of MK5 by its interaction partners causes the redistribution of MK5 from nucleus to cytoplasm [42, 50, 72, 73, 74]. TLK1‐mediated phosphorylation on Ser354 residue, which lies on the NLS domain of MK5, may mask the NLS and promote its nuclear export to exert its functions on motility enhancement.

## 5 Conclusion

Implementation of ADT and novel antiandrogens has made an impact in the early survival of PCa patients, but the presence of metastasis remains a significant poor prognostic indicator. Inhibition of metastatic dissemination seems to be the first and foremost challenge in the treatment of mCRPC patients. We identified a novel signalling axis (TLK1‐MK5), which is active in the majority of the PCa cell lines and demonstrated that disruption of this signalling significantly reduces PCa cell migration. Further research is needed to determine whether the inhibition of TLK1‐MK5 signalling can reduce PCa metastasis in xenograft mouse model using TLK1 and/or MK5 inhibitor. Successful completion of such work may result in the application of these inhibitors in combination with other treatment strategies to delay PCa metastatic burden and lethal progression.

## Conflict of interest

The authors declare that no conflict of interest exist.

### Peer Review

The peer review history for this article is available at https://publons.com/publon/10.1002/1878‐0261.13183.

## Author contributions

MIK and VS generated most of the data. JK provided expertise and analysis of the TMA and mouse IHC studies. MIK designed some of the research, wrote much of the paper and conducted the analysis of the TCGA data. ADB directed and designed the research and wrote portions of the paper.

## Supporting information


**Fig. S1**. MK5 overexpression and image representation of the scratch wound repair assay among different MEF cells.
**Fig. S2**. MK5 rescue and TLK1 overexpression and image representation of the scratch wound repair assay among different MEF cells.
**Fig. S3**. Image representation of 3D chemotactic trans‐well migration assay among different MEF cells.
**Fig. S4**. 3D chemotactic trans‐well migration assay among different MEF cells using Matrigel coating.
**Fig. S5**. 3D chemotactic trans‐well migration assay among different MEF cells using collagen coating.
**Fig. S6**. Image representation of the proliferation assay between MK5 rescued and MK5^−/−^ MEF cells.
**Fig. S7**. MK5 or kinase dead TLK1 overexpression and image representation of the scratch wound repair assay among different LNCaP cells.
**Fig. S8**. TLK1 knockdown and image representation of scratch wound repair assays among different MEF cells.
**Fig. S9**. Cell proliferation determination among MEF MK5^−/−^ GFP‐MK5 cells by MTS assay.
**Fig. S10**. Wound healing rate between treated or untreated MEF MK5^−/−^ cells determined by scratch wound repair assay.
**Fig. S11**. Wild‐type or mutant variant of MK5 reconstitution and image representation of the scratch wound repair assay among different MEF MK5^−/−^ cells.
**Fig. S12**. Recombinant protein purification and MK5 kinase activity determination by ADP hunter assay.
**Fig. S13**. Spectral analysis of MK5 phosphoresidue determination by MS.
**Fig. S14**. MK5 phosphopeptide determination by MS.
**Fig. S15**. Schematic diagram of MK5 domains with TLK1 mediated phosphoresidues.
**Fig. S16**. Nucleocytoplasmic shuttling of GFP‐MK5 upon TLK1 overexpression in HEK 293 cells.
**Fig. S17**. Image representation of the scratch wound repair assays among different PCa cell lines treated with different concentration of GLPG0259.
**Fig. S18**. Kaplan‐Meier survival plot of PRAD patients based on TLK1 expression and Gleason scores.

**Table S1**. Peptides containing unique phosphorylated sites detected in the TLK1‐MK5 samples.

**Table S2**. Layout of PCa TMA.
**Table S3**. Clinicopathological features of the TMA tumour samples.

## Data Availability

All data generated or analysed during this study and its supplementary information files are included in this published article. Cell lines and most reagents generated for this study are available upon request.
