# Supplementary material for: TLK1‐mediated MK5‐S354 phosphorylation drives prostate cancer cell motility and may signify distinct pathologies
Source: Mol Oncol. 2022 Feb 3;16(13):2537–57. doi: 10.1002/1878-0261.13183 (PMC9251878; doi:10.1002/1878-0261.13183)

Supplementary Figure: S1

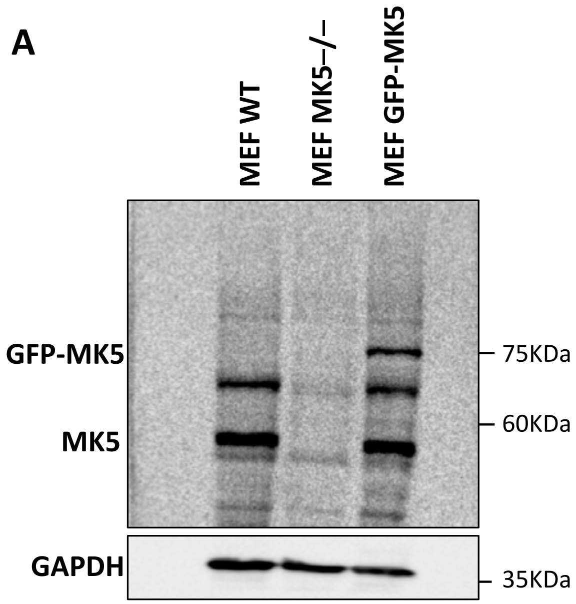

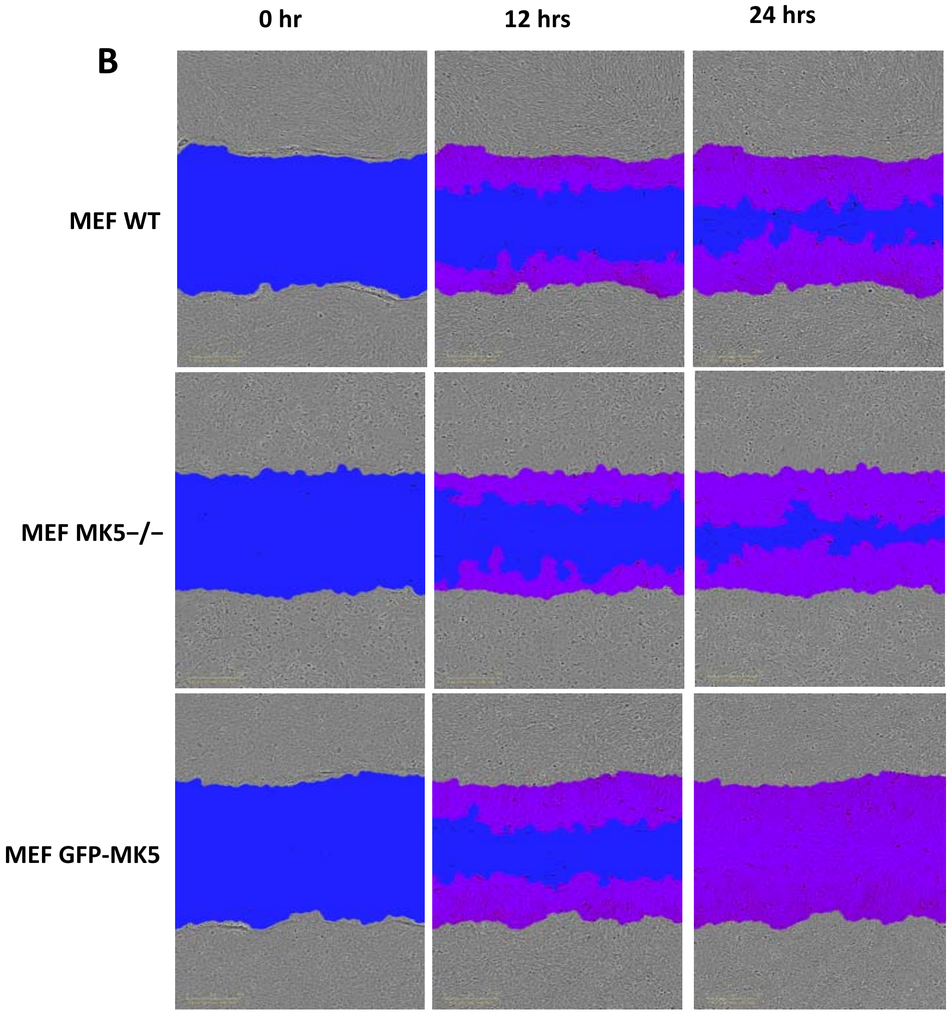

Supplementary Figure: S2

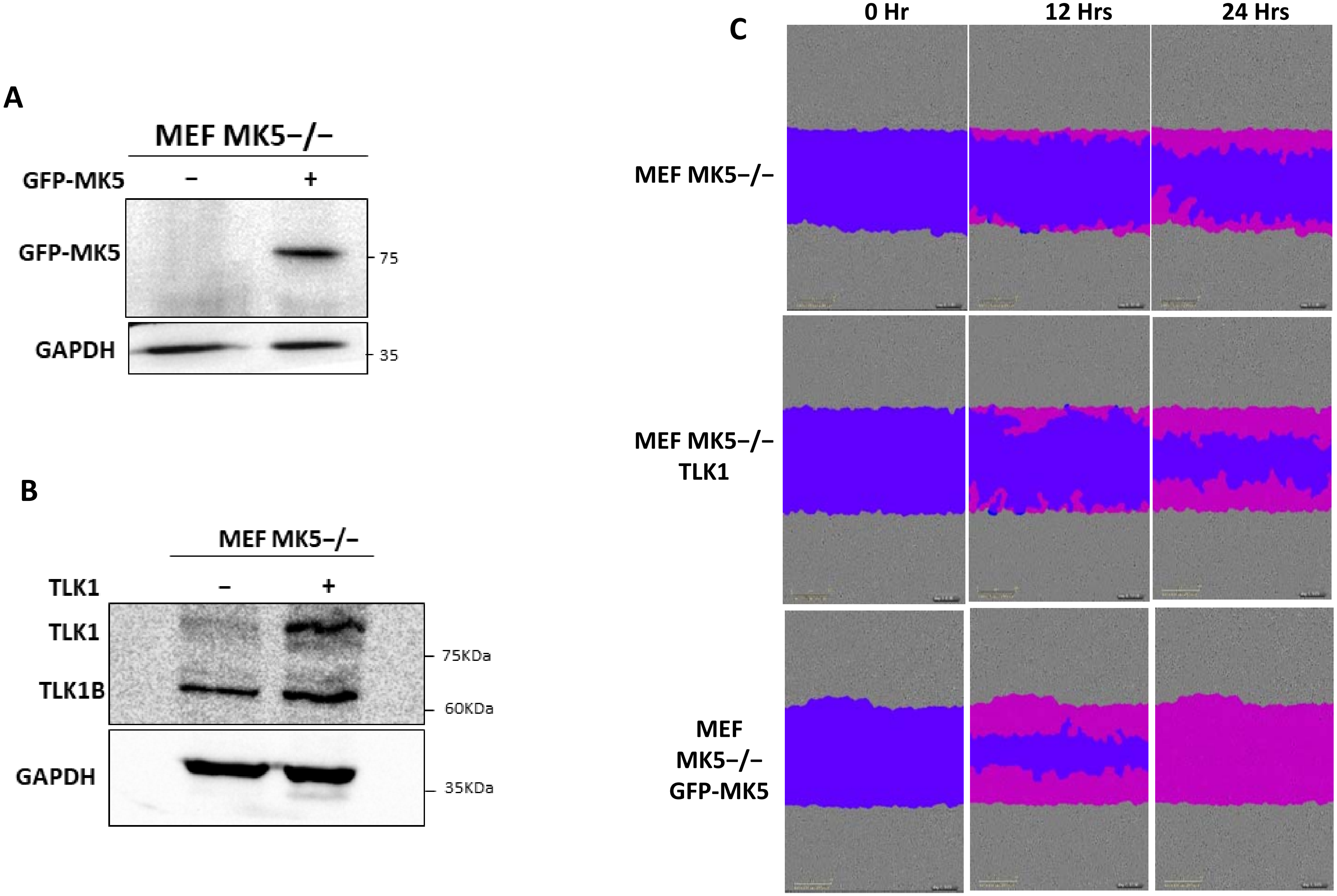

Supplementary Figure: S3

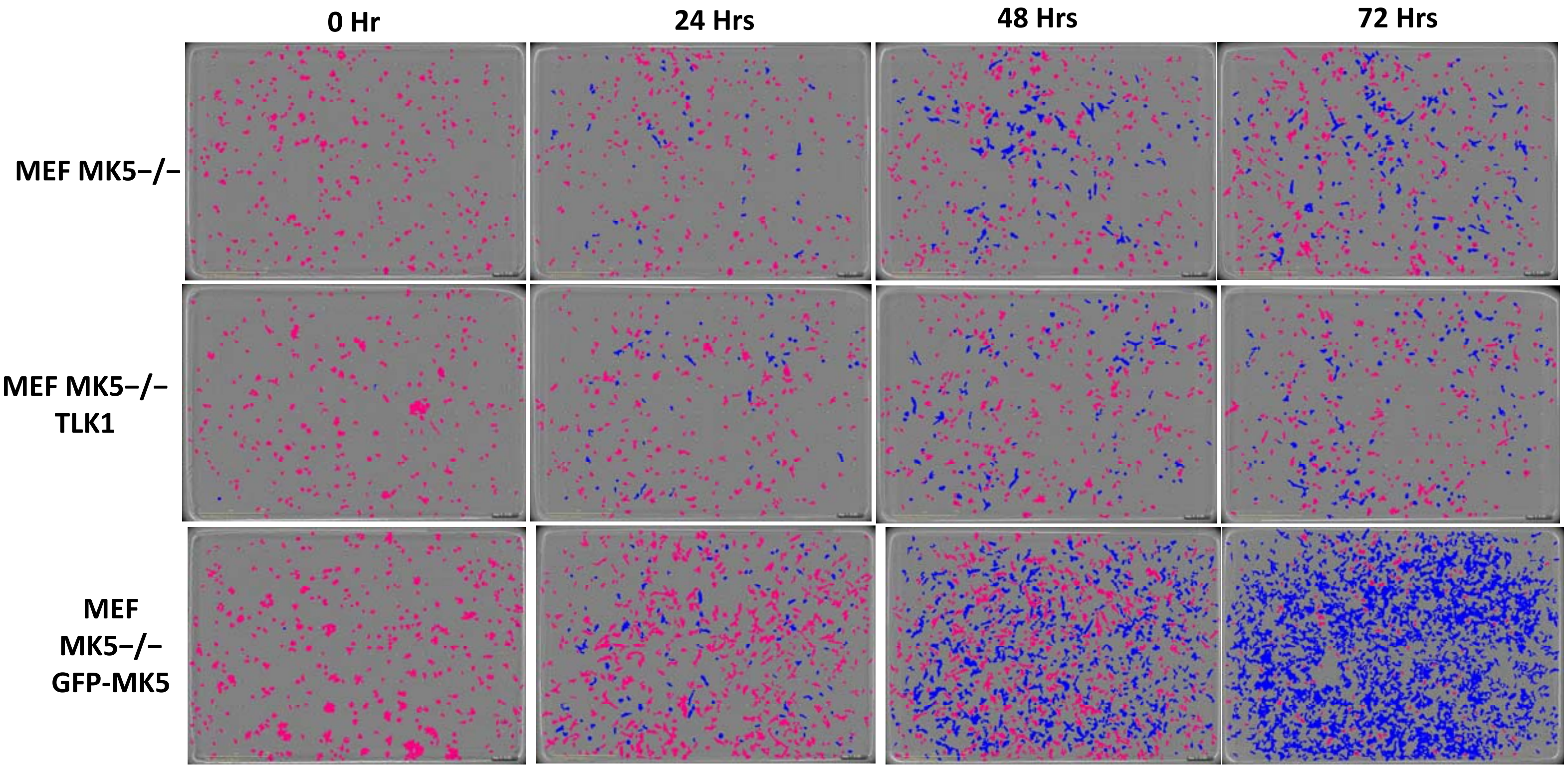

Supplementary Figure: S4

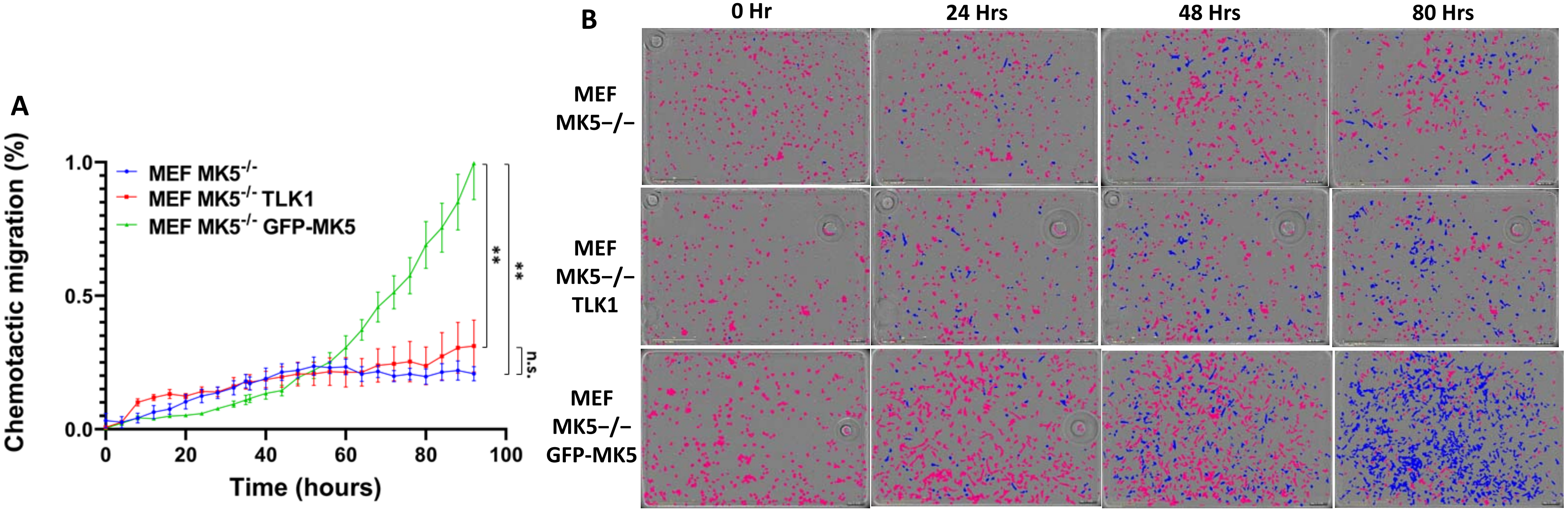

Supplementary Figure: S5

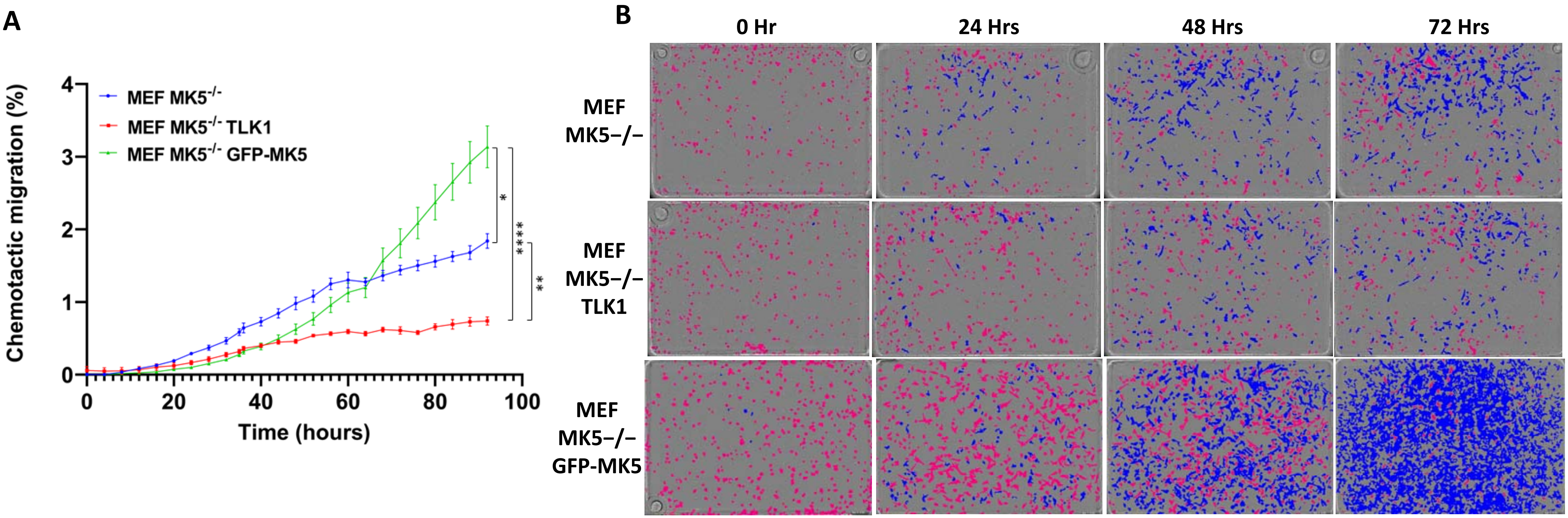

Supplementary Figure: S6

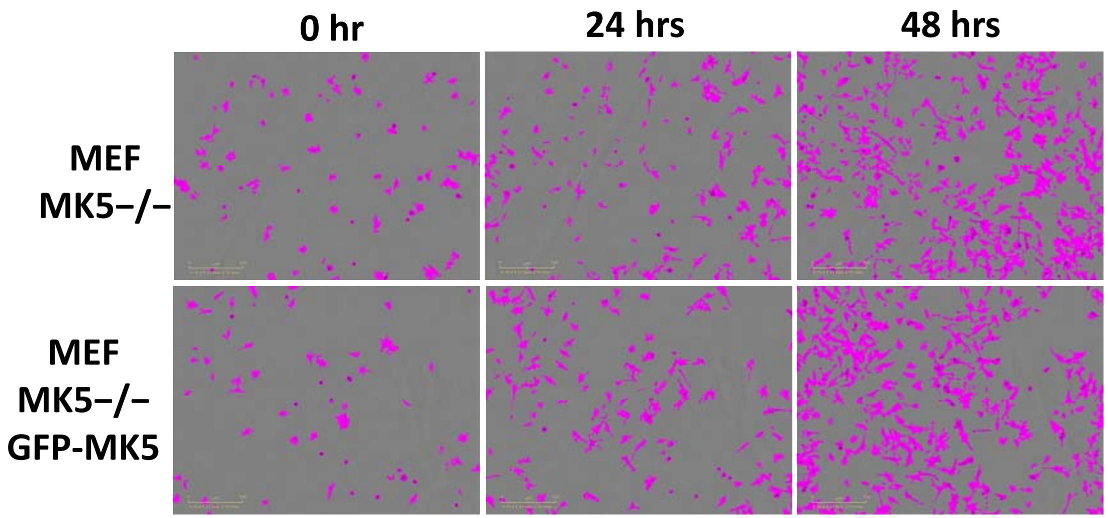

Supplementary Figure: S7

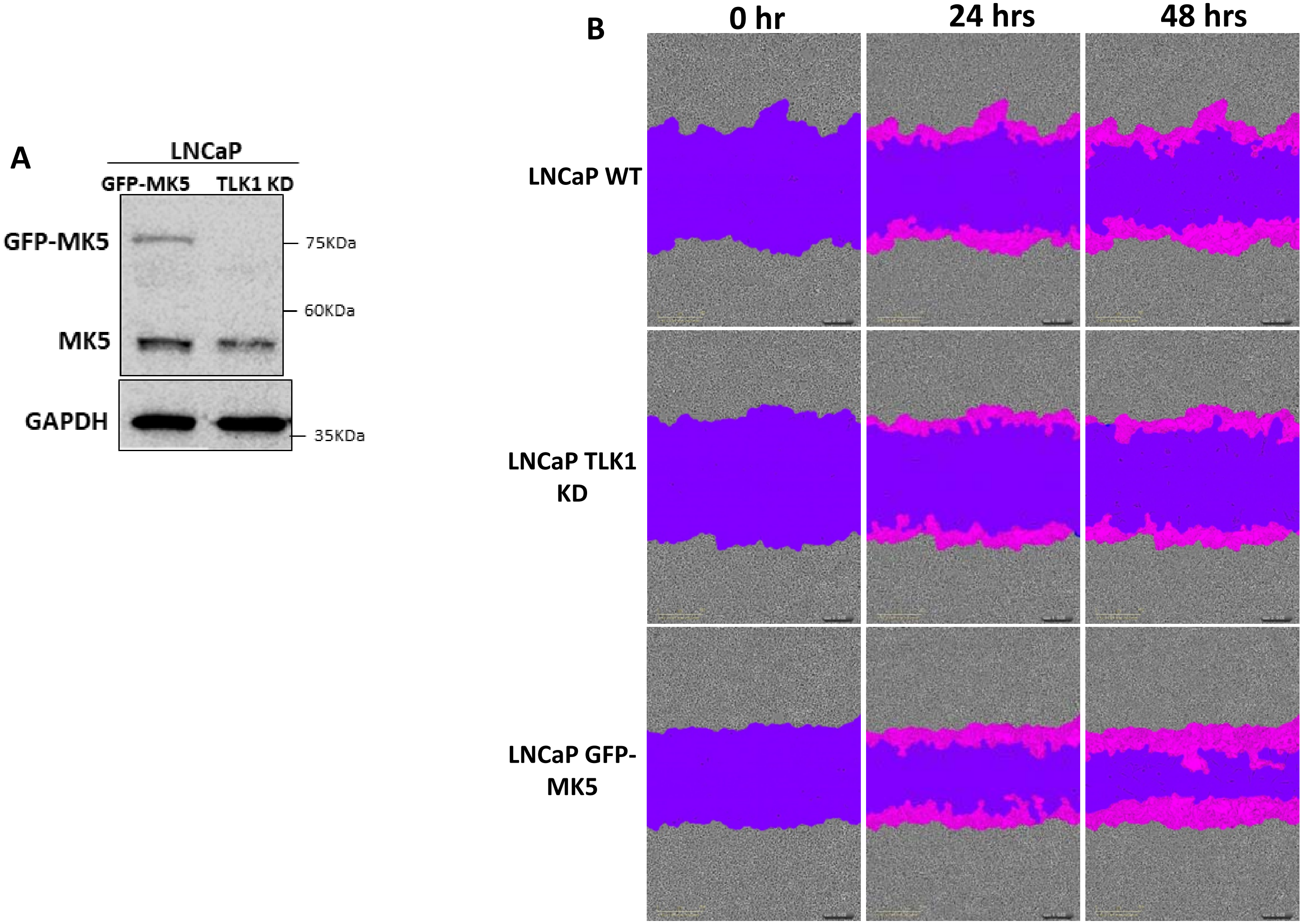

Supplementary Figure: S8

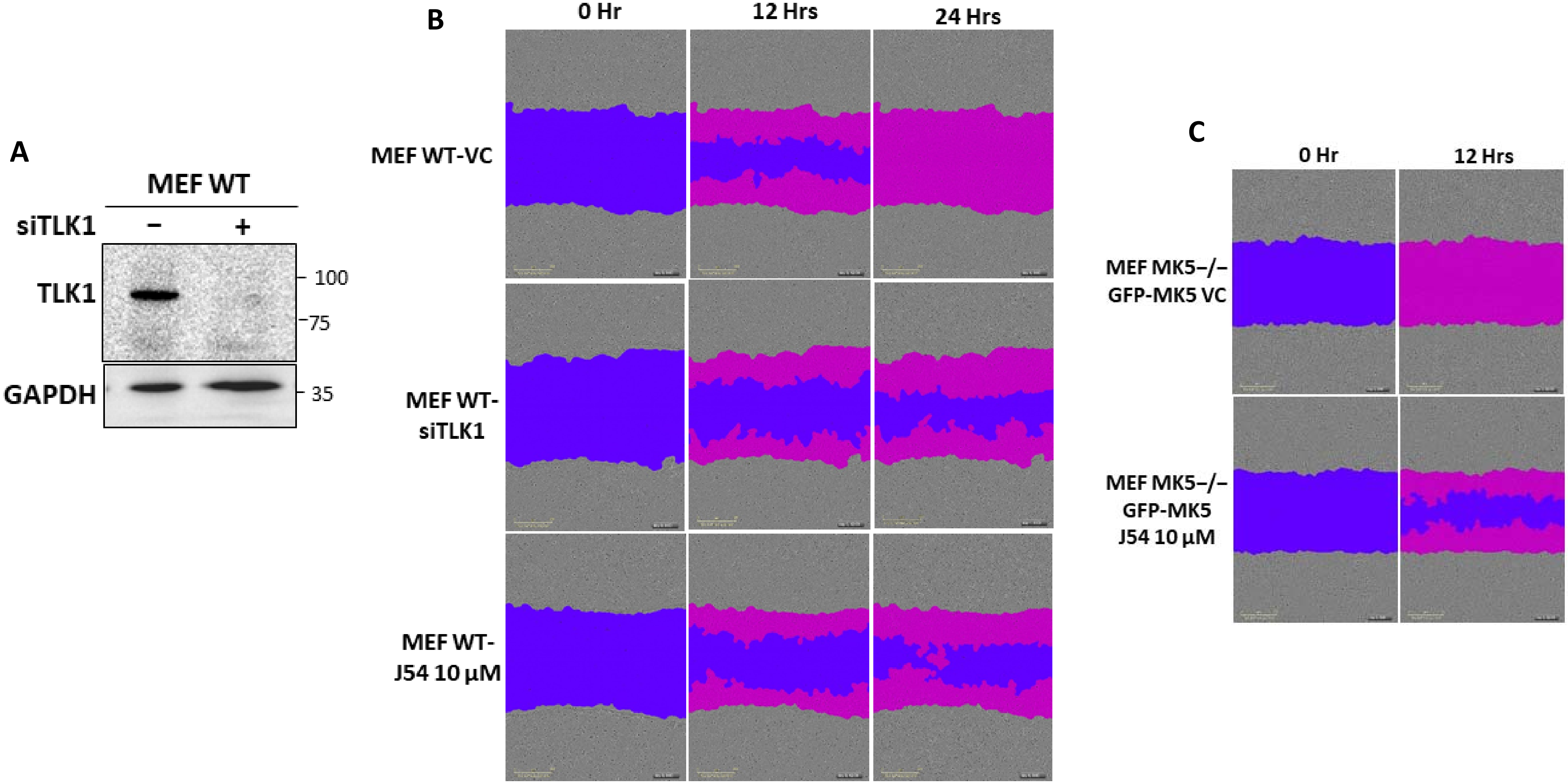

Supplementary Figure: S9

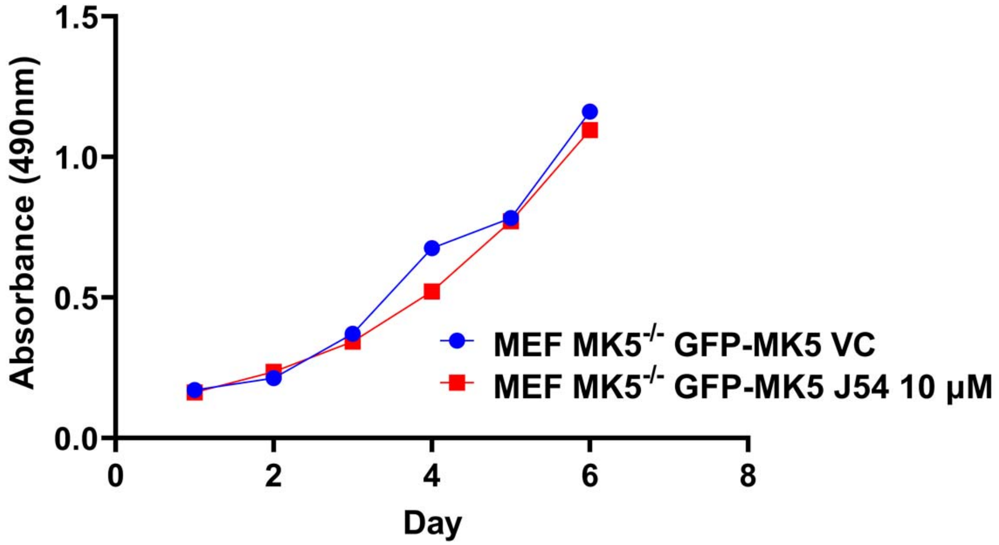

Supplementary Figure: S10

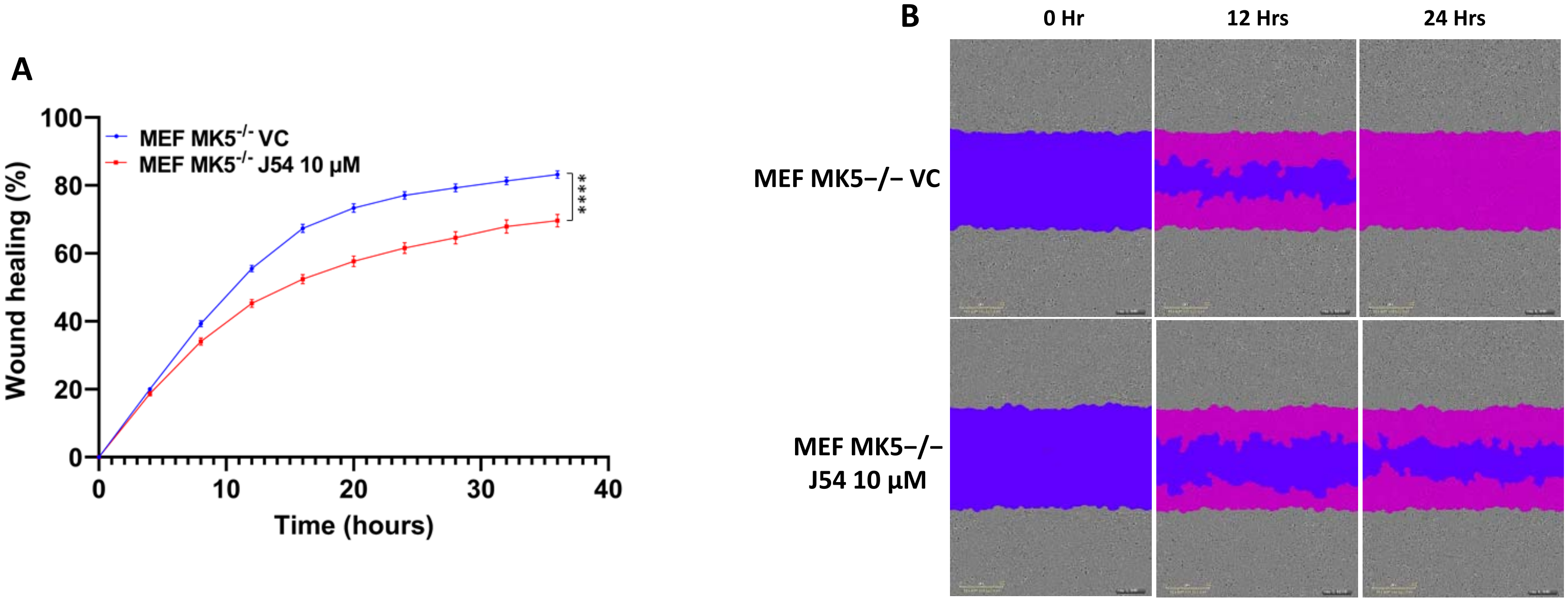

Supplementary Figure: S11

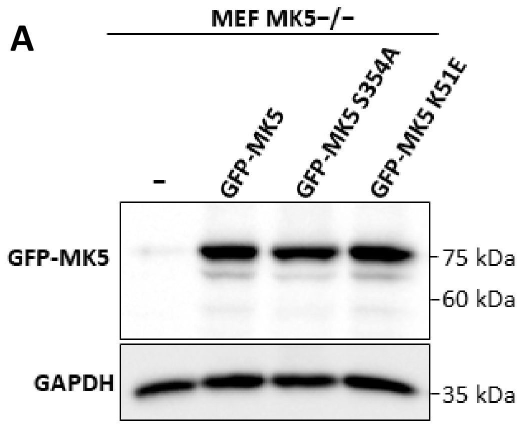

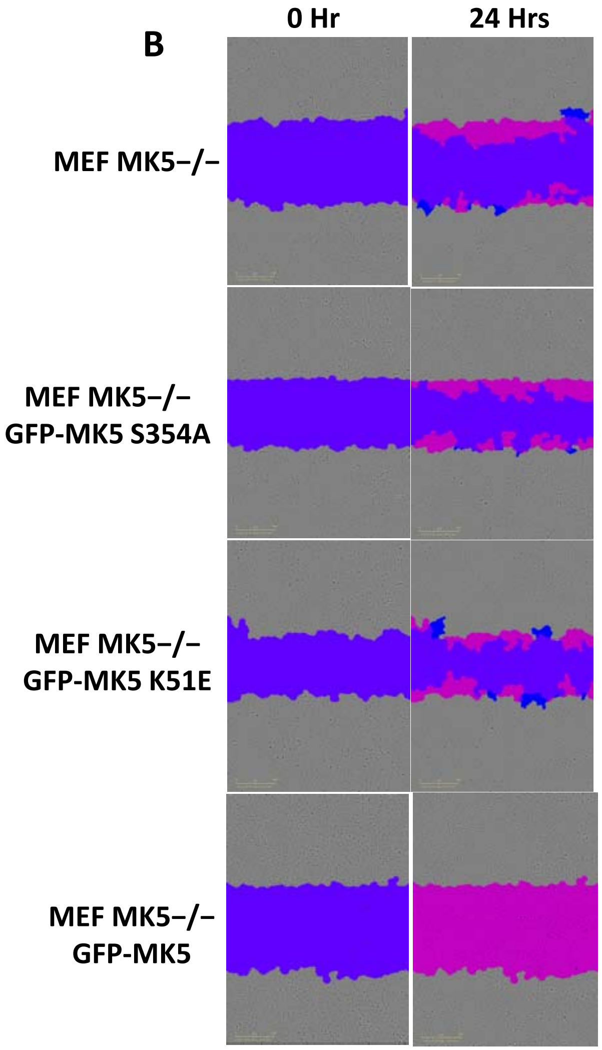

Supplementary Figure: S12

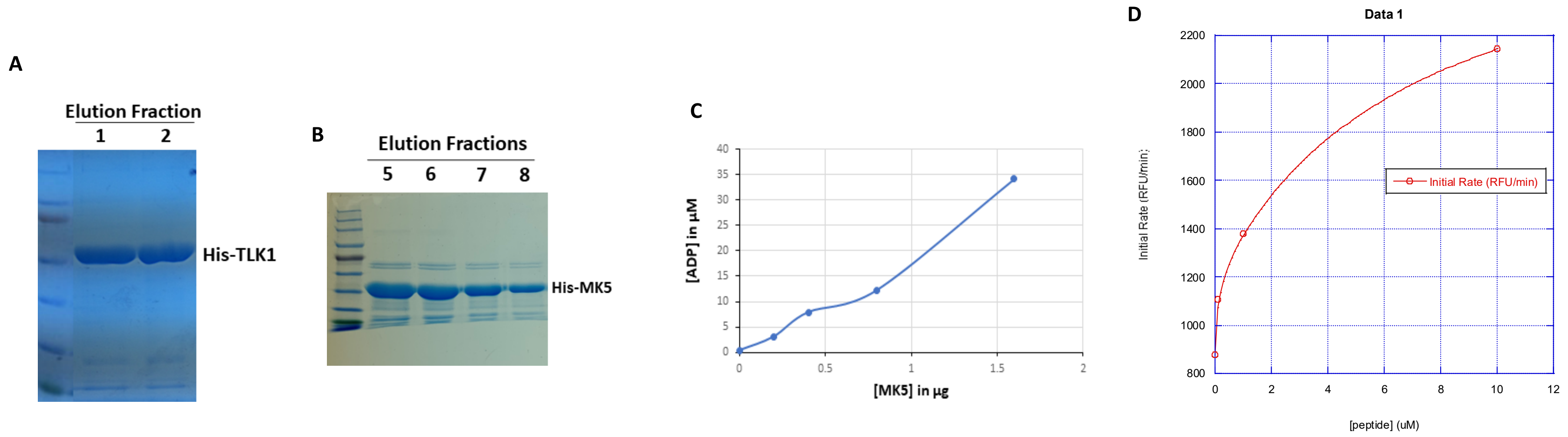

Supplementary Figure: S13

A

MS/MS spectrum of the phosphor-peptide $D_{148}LKPENLLFKDNSLDAPVK_{166}$ to determine pS160

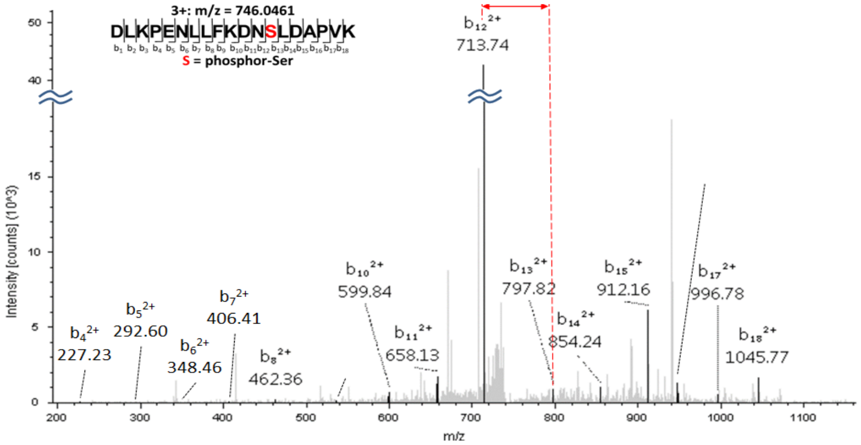

B

MS/MS spectrum of the phosphor-peptide $V_{347}SLKPLHSVNNPILR_{362}$ to determine pS354

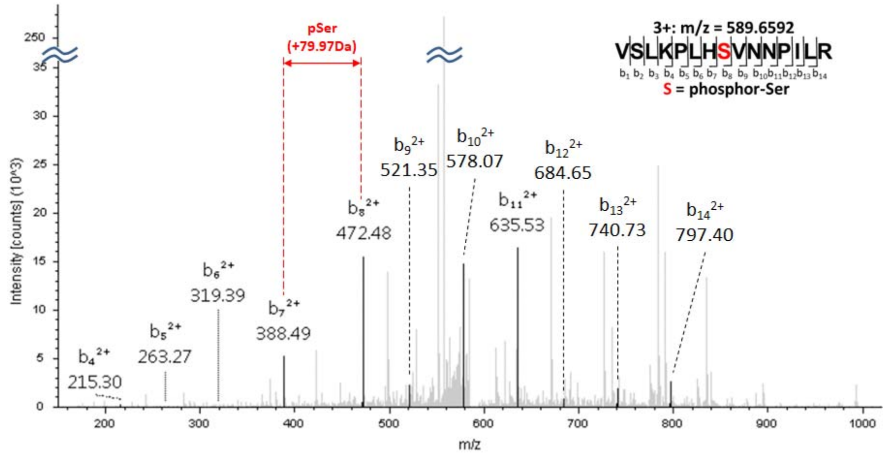

C

MS/MS spectrum of the phosphor-peptide $D_{372}SVYIHDHENGAEDSNVALEK_{392}$ to determine pS386

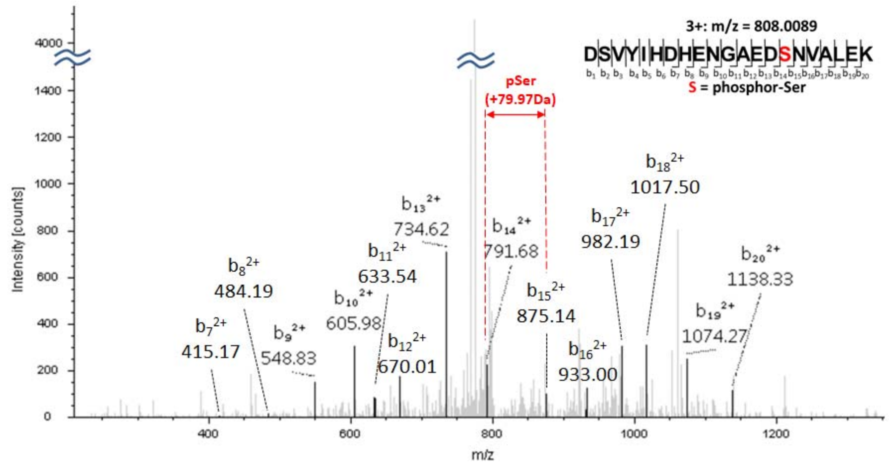

Supplementary Figure: S14

MK5-Control

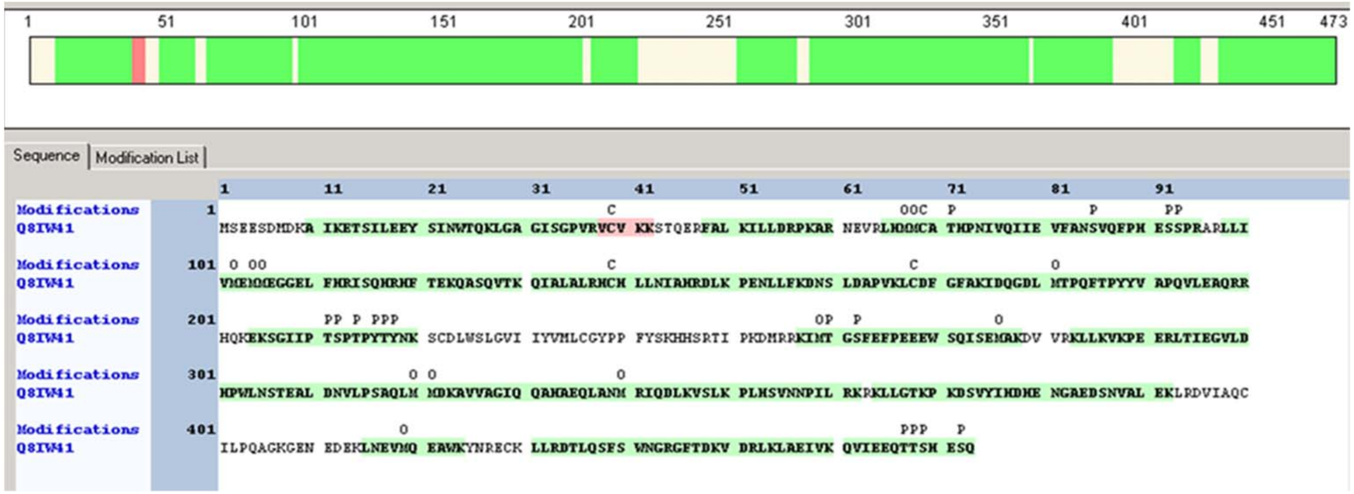

New phosphorylated sites detected in MK5-plus sample are marked with red circles.

MK5+TLK1B

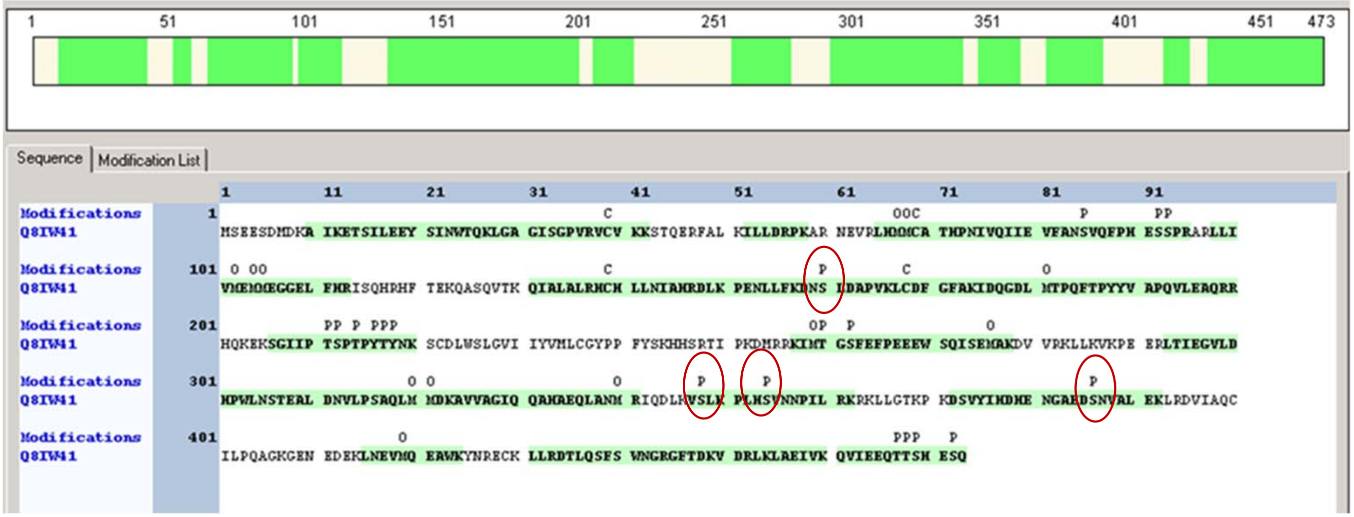

Supplementary Figure: S15

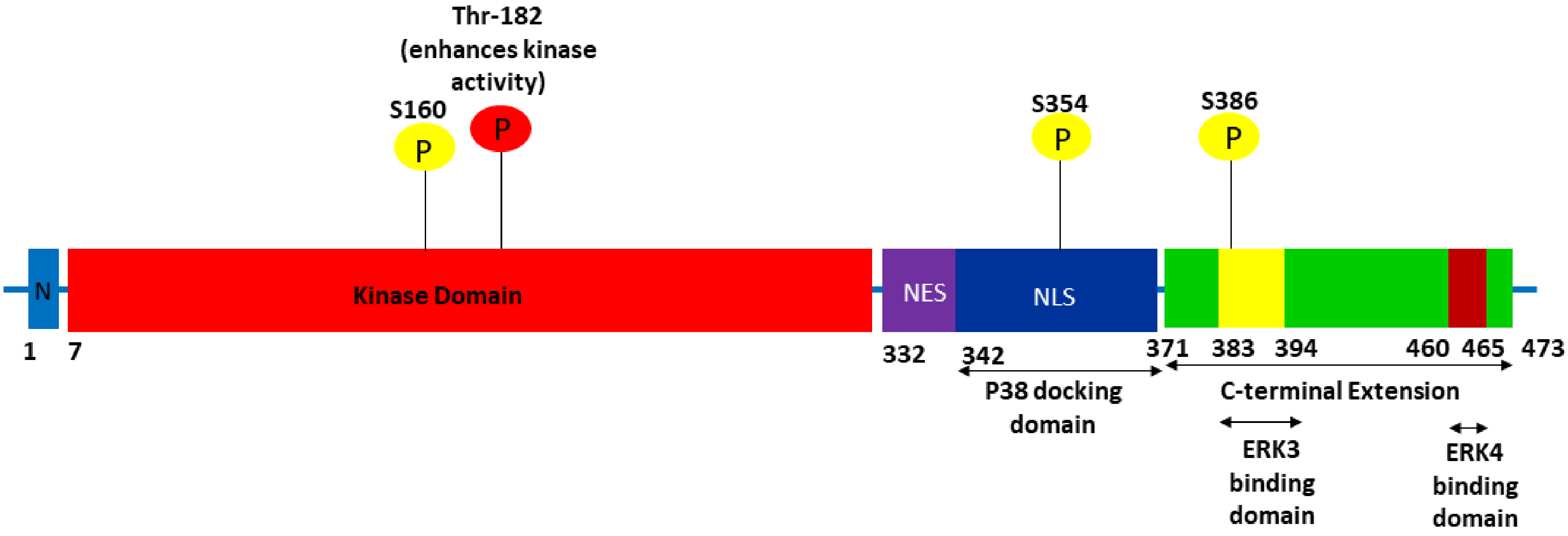

Supplementary Figure: S16

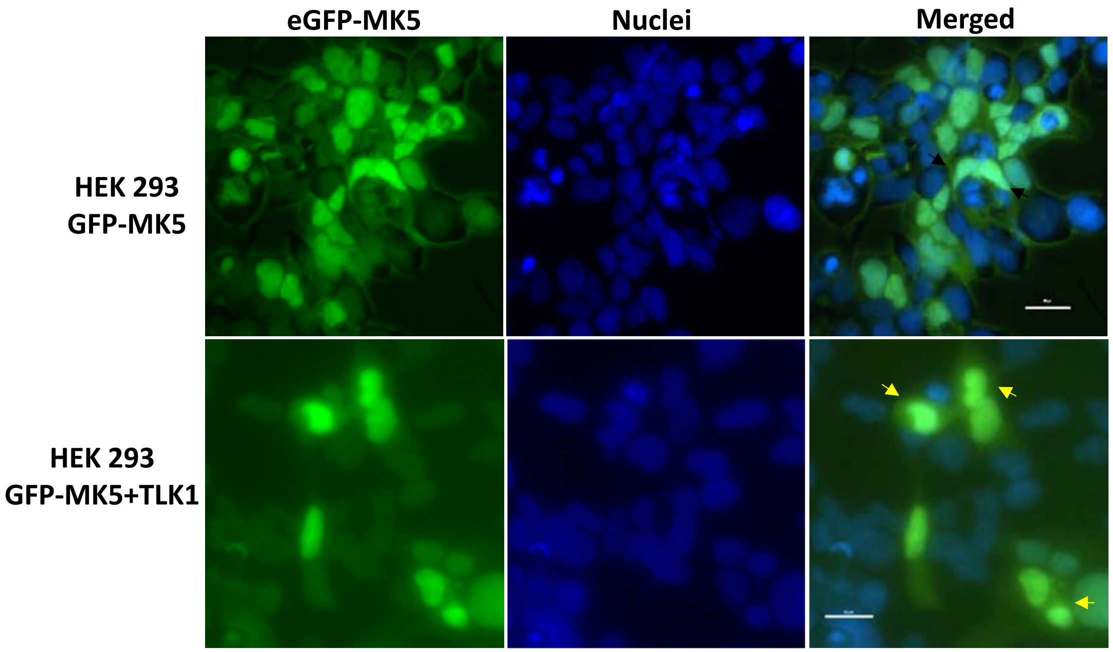

Supplementary Figure: S17

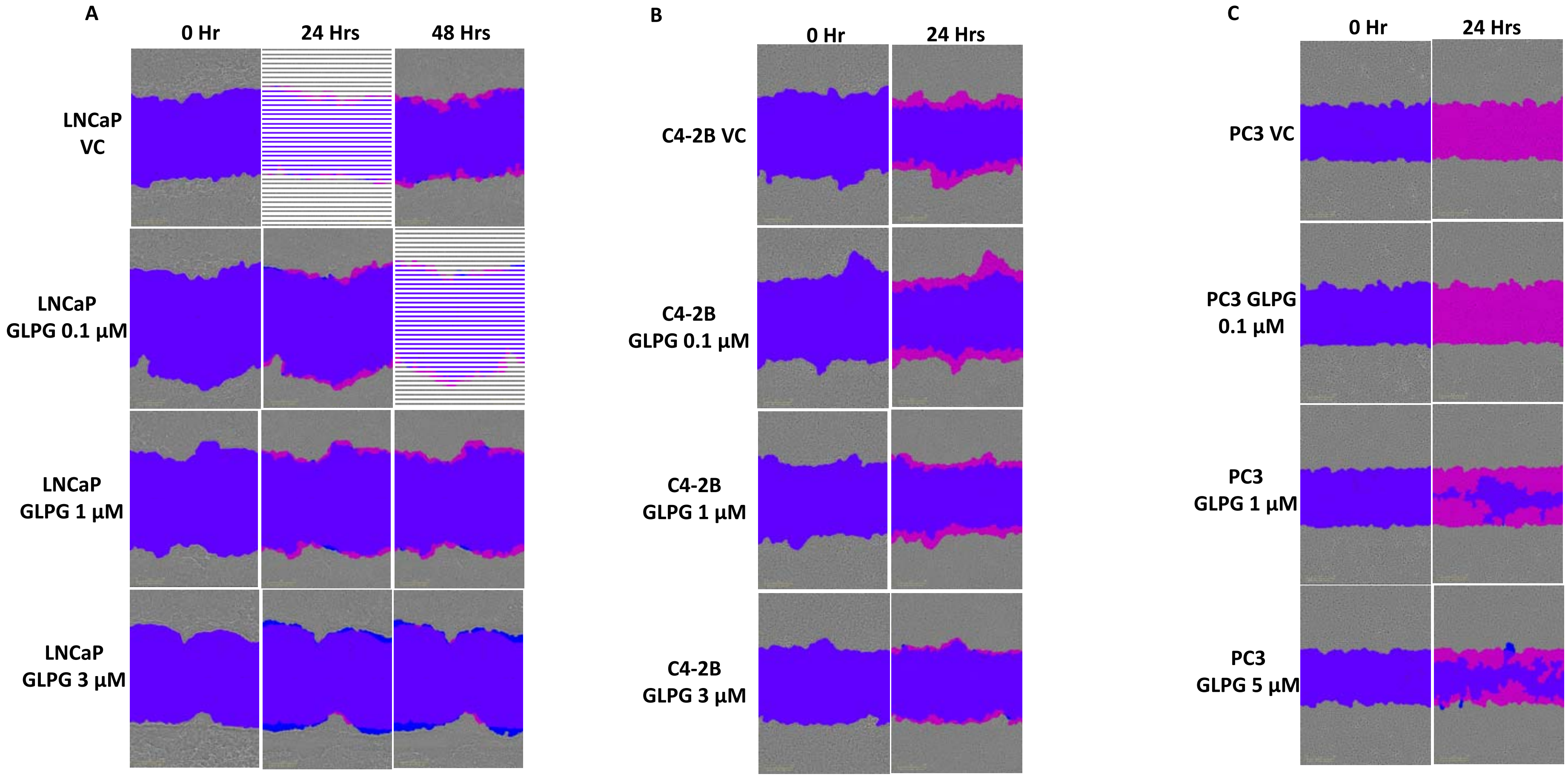

Supplementary Figure: S17 (continued)

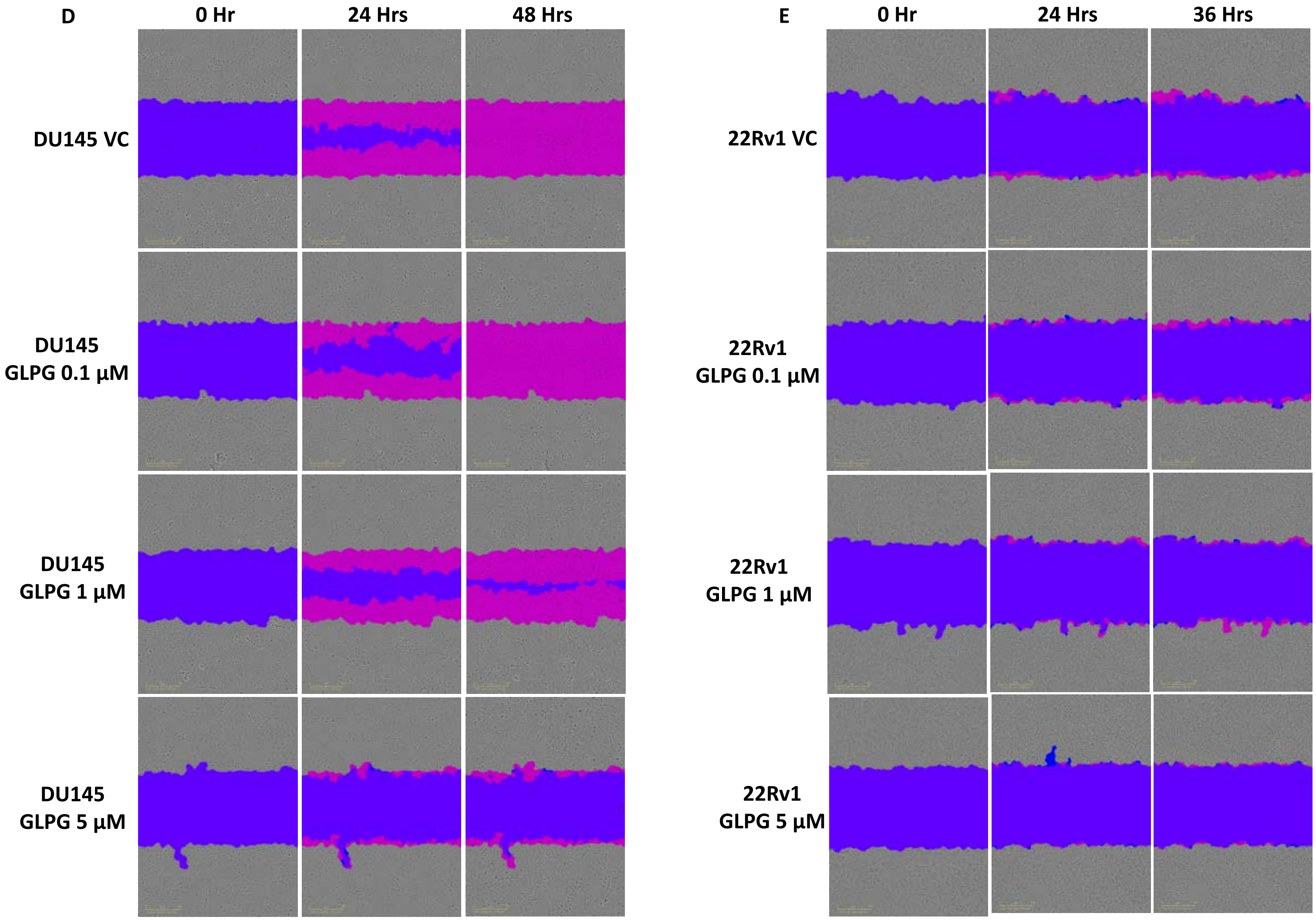

Supplementary Figure: S18

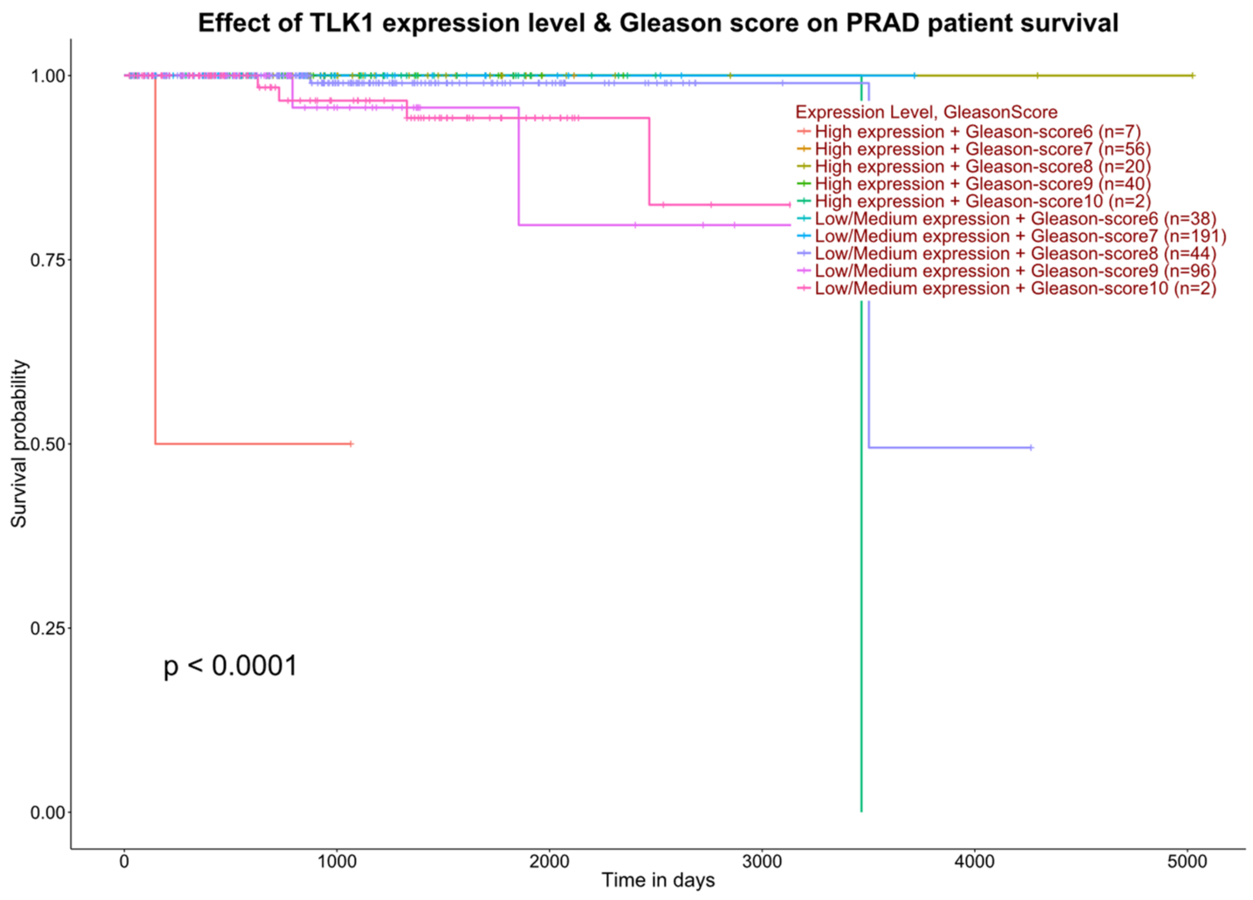

Supplement: Supplementary file 1 — Fig. S1. MK5 overexpression and image representation of the scratch wound repair assay among different MEF cells. Fig. S2. MK5 rescue and TLK1 overexpression and image representation of the scratch wound repair assay among different MEF cells. Fig. S3. Image representation of 3D chemotactic trans‐well migration assay among different MEF cells. Fig. S4. 3D chemotactic trans‐well migration assay among different MEF cells using Matrigel coating. Fig. S5. 3D chemotactic trans‐well migration assay among different MEF cells using collagen coating. Fig. S6. Image representation of the proliferation assay between MK5 rescued and MK5−/− MEF cells. Fig. S7. MK5 or kinase dead TLK1 overexpression and image representation of the scratch wound repair assay among different LNCaP cells. Fig. S8. TLK1 knockdown and image representation of scratch wound repair assays among different MEF cells. Fig. S9. Cell proliferation determination among MEF MK5−/− GFP‐MK5 cells by MTS assay. Fig. S10. Wound healing rate between treated or untreated MEF MK5−/− cells determined by scratch wound repair assay. Fig. S11. Wild‐type or mutant variant of MK5 reconstitution and image representation of the scratch wound repair assay among different MEF MK5−/− cells. Fig. S12. Recombinant protein purification and MK5 kinase activity determination by ADP hunter assay. Fig. S13. Spectral analysis of MK5 phosphoresidue determination by MS. Fig. S14. MK5 phosphopeptide determination by MS. Fig. S15. Schematic diagram of MK5 domains with TLK1 mediated phosphoresidues. Fig. S16. Nucleocytoplasmic shuttling of GFP‐MK5 upon TLK1 overexpression in HEK 293 cells. Fig. S17. Image representation of the scratch wound repair assays among different PCa cell lines treated with different concentration of GLPG0259. Fig. S18. Kaplan‐Meier survival plot of PRAD patients based on TLK1 expression and Gleason scores. [file MOL2-16-2537-s003.pdf]
