# Supplementary material for: TLK1‐mediated MK5‐S354 phosphorylation drives prostate cancer cell motility and may signify distinct pathologies
Source: Mol Oncol. 2022 Feb 3;16(13):2537–57. doi: 10.1002/1878-0261.13183 (PMC9251878; doi:10.1002/1878-0261.13183)
Supplement: Supplementary file 2 — Table S1. Peptides containing unique phosphorylated sites detected in the TLK1‐MK5 samples. [file MOL2-16-2537-s002.pdf]

Supplementary Table: 1

Peptide	Phosphorylation Sites	MOWSE scores
D_{372}SVYIHDHENGAEDSNVALEK$_{392}$	S386 (Phosp)	44
V_{347}SLKPLHSVNNPILR$_{362}$	S354 (Phosp)	32
D_{148}LKPENLLFKDNSLDAPVK$_{166}$	S160 (Phosp)	25
